# Molecular stratification of endometrioid ovarian carcinoma predicts clinical outcome

**DOI:** 10.1038/s41467-020-18819-5

**Published:** 2020-10-05

**Authors:** Robert L. Hollis, John P. Thomson, Barbara Stanley, Michael Churchman, Alison M. Meynert, Tzyvia Rye, Clare Bartos, Yasushi Iida, Ian Croy, Melanie Mackean, Fiona Nussey, Aikou Okamoto, Colin A. Semple, Charlie Gourley, C. Simon Herrington

**Affiliations:** 1 Nicola Murray Centre for Ovarian Cancer Research, Cancer Research UK Edinburgh Centre, MRC Institute of Genetics and Molecular Medicine, University of Edinburgh, Edinburgh, UK; 2 MRC Human Genetics Unit, MRC Institute of Genetics and Molecular Medicine, University of Edinburgh, Edinburgh, UK; 3 The Jikei University School of Medicine, Tokyo, Japan; 4 Edinburgh Cancer Centre, Western General Hospital, Edinburgh, UK

**Keywords:** Cancer, Gynaecological cancer, Ovarian cancer

## Abstract

Endometrioid ovarian carcinoma (EnOC) demonstrates substantial clinical and molecular heterogeneity. Here, we report whole exome sequencing of 112 EnOC cases following rigorous pathological assessment. We detect a high frequency of mutation in *CTNNB1* (43%), *PIK3CA* (43%), *ARID1A* (36%), *PTEN* (29%), *KRAS* (26%), *TP53* (26%) and *SOX8* (19%), a recurrently-mutated gene previously unreported in EnOC. *POLE* and mismatch repair protein-encoding genes were mutated at lower frequency (6%, 18%) with significant co-occurrence. A molecular taxonomy is constructed, identifying clinically distinct EnOC subtypes: cases with *TP53* mutation demonstrate greater genomic complexity, are commonly FIGO stage III/IV at diagnosis (48%), are frequently incompletely debulked (44%) and demonstrate inferior survival; conversely, cases with *CTNNB1* mutation, which is mutually exclusive with *TP53* mutation, demonstrate low genomic complexity and excellent clinical outcome, and are predominantly stage I/II at diagnosis (89%) and completely resected (87%). Moreover, we identify the WNT, MAPK/RAS and PI3K pathways as good candidate targets for molecular therapeutics in EnOC.

## Abstract (web summary)

The molecular classification of endometroid ovarian carcinomas (EnOC) has not been established, preventing the development of stratified therapeutic approaches. Here the authors characterise the molecular landscape of EnOC by whole exome sequencing, identifying clinically distinct disease subtypes.

## Introduction

Ovarian carcinomas (OC) are a heterogeneous group of malignancies comprising five core histological types, each with distinct pathological characteristics, molecular landscapes and clinical behaviour^1,2^. Endometrioid OC (EnOC) accounts for approximately 10% of all OC, with the majority of cases diagnosed as low grade, early stage disease with excellent clinical outcome^3–5^.

Currently, the management of EnOC follows the historic one-size-fits-all approach of aggressive cytoreductive surgery with adjuvant platinum–taxane chemotherapy for patients with disease that has progressed beyond the ovary/fallopian tube. By contrast, routine molecular stratification of care is emerging in other OC types, most notably with the advent of poly(adenosine diphosphate-ribose) polymerase (PARP) inhibitor therapy^6,7^.

Targeted sequencing approaches have identified *PTEN, ARID1A, PIK3CA, KRAS, CTNNB1*, and genes encoding mismatch repair (MMR) proteins as frequently mutated in relatively small cohorts of EnOC^8–10^, reminiscent of endometrioid endometrial carcinoma (EnEC)^11^, with a *TP53* mutation (*TP53*m) rate markedly lower than their high grade serous OC (HGSOC) counterparts^12^. Recent whole genome sequencing of a small EnOC case series has recapitulated these findings and identified a small proportion of EnOC with extensive copy number alterations (CNAs) more akin to the genomic instability demonstrated by HGSOC^13^.

The majority of EnOC are believed to arise from endometriosis^1^, and most grade 1 and 2 (low grade) EnOC display a classical immunohistochemical (IHC) profile comprising Wilms’ tumour 1 (WT1) negativity, wild-type tumour protein p53 (p53) expression, and oestrogen receptor (ER) positivity^4^. These classical low grade EnOC bear close histological resemblance to EnEC^14^.

Grade 3 (high grade) EnOC can be challenging to differentiate from HGSOC on the basis of morphology alone^4,14^. In particular, HGSOC demonstrating the solid, pseudo-endometrioid and/or transitional-cell-like (SET) histological pattern, which may be associated with *BRCA1* mutations^15^, represent a population easily misclassified as EnOC. Indeed, it is now recognised that many historically diagnosed high grade EnOC in fact represent HGSOC, supported by transcriptomic studies demonstrating that a proportion of high grade EnOC cluster with HGSOC^16–19^. As such, true high grade EnOC are increasingly rare, representing only around 5–19 % of EnOC cases^4,9,20,21^; these patients reportedly experience poor clinical outcome, in contrast to their low grade counterparts^3,21^.

To date, the overwhelming body of clinical and molecular EnOC characterisation has been confounded by the inclusion of historically misclassified HGSOC. Mutational analyses performed by more recent studies have either been applied to low grade EnOC alone^22^, or lack information on grade or diagnostic criteria used^8,23^ and have ubiquitously analysed small patient cohorts with insufficient power to confidently correlate molecular events with patient outcome. As a result, the molecular landscape of EnOC, in particular high grade EnOC, is poorly defined.

WT1 IHC is a useful tool to discriminate high grade EnOC (WT1 negative) from HGSOC (WT1 positive), reducing interobserver variation^16,20,24–27^. Here, we perform molecular characterisation of contemporarily defined EnOC with the use of IHC for WT1. We perform whole exome sequencing (WES) to define the genomic landscape of EnOC, including high grade EnOC, in a sizeable cohort of otherwise unselected patients. We identify subtypes of EnOC that display distinct clinical behaviour, constructing a step-wise taxonomy for EnOC classification based on mutation status of *TP53* and *CTNNB1*. *TP53*m cases, characterised by greater genomic complexity and frequent CNA events, demonstrate poor survival; conversely, cases with *CTNNB1* mutation (*CTNNB1*m)—which occurs mutually exclusively with *TP53*m—are of low genomic complexity with few CNA events and demonstrate excellent long-term survival. The remaining cases represent a subtype with intermediate prognosis.

## Results

### Clinical characteristics

**Fig. 1:**
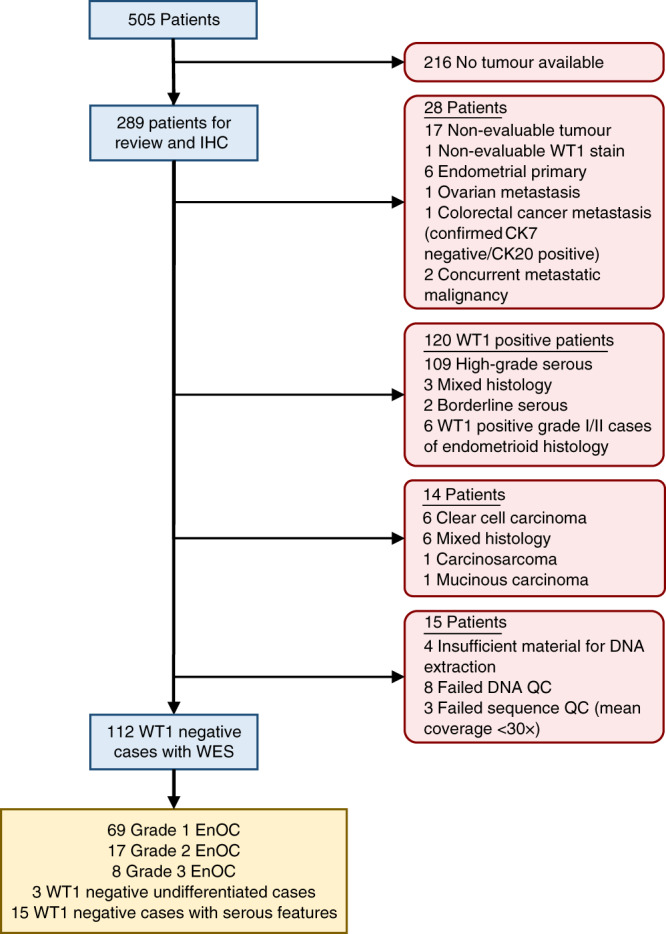
Flow diagram identifying endometrioid ovarian carcinoma cases for whole exome sequencing. IHC immunohistochemistry, WES whole exome sequencing, EnOC endometrioid ovarian carcinoma, WT1 Wilms’ tumour 1, QC quality control.

Of 289 historically diagnosed EnOC cases identified with available tumour material, 112 WT1 negative cases were characterised by WES following rigorous pathology review (Fig. 1). The clinicopathological characteristics of these patients are shown in Table 1. The majority of patients presented with stage I or II disease (78.2%, 86 of 110 evaluable cases; 2 unknown stage); 27 stage I/II cases received no adjuvant chemotherapy. Nineteen cases (17.0%) had concurrent endometrial cancer diagnosis. The median follow-up time was 13.0 years. Five-year disease-specific survival (DSS) and progression-free survival (PFS) across the cohort was 72.8% (95% CI 64.8–81.8%) and 68.5% (95% CI 60.2–77.9%).

**Table 1:** Clinical characteristics of the 112 endometrioid ovarian carcinoma cases.

	*N*/median	%/range
Cases	112	
Age (years)	58.5	28–88
BMI (kg per square metre height)	25.4	18.0–44.0
Concurrent endometrial cancer^a^	19	17.0
Endometrioisis^b^	39	34.8
*Grade*		
Grade 1 EnOC	69	61.6
Grade 2 EnOC	17	15.2
Grade 3 EnOC	8	7.1
Undifferentiated	3	2.7
Serous-like features	15	13.4
*Vital status at last follow-up*		
Deceased, ovarian cancer	35	31.3
Deceased, other causes	19	17.0
Alive	58	51.8
*Period of diagnosis*		
1980s	17	15.2
1990s	44	39.3
2000s	32	28.6
2010s	19	17.0
*FIGO stage at diagnosis*		
I	47	42.7
II	39	35.5
III	15	13.6
IV	9	8.2
NA	2	–
*Primary debulking status*		
Zero macroscopic RD	82	78.1
Macroscopic RD	23	21.9
NA	7	–
*Adjuvant chemotherapy stage I/II* (*n* = *86*)		
Single-agent platinum	31	36.5
Platinum–taxane combination	17	20.0
Other platinum combination	3	3.5
Other chemotherapy regime	7	8.2
No adjuvant chemotherapy	27	31.8
NA	1	–
*Adjuvant therapy stage III/IV (n* = *24)*		
Single-agent platinum	14	58.3
Platinum–taxane combination	2	8.3
Other platinum combination	2	8.3
Other chemotherapy regime	1	4.2
No adjuvant chemotherapy	5	20.8

*BMI* body mass index, *EnOC* endometrioid ovarian carcinoma, *NA* not available, *RD* residual disease, *FIGO* International Federation of Obstetrics and Gynecology.

^a^Documented on the Edinburgh Ovarian Cancer Database.

^b^Documented on the diagnostic pathology report or identified from reviewed archival tissue.

### Genomic landscape of endometrioid ovarian carcinoma

**Fig. 2:**
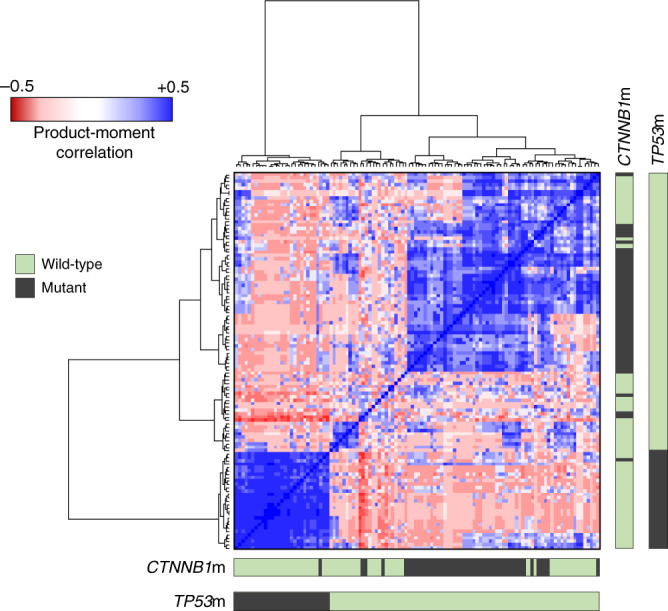
Unsupervised clustering of endometrioid ovarian carcinomas by patterns of mutation. Product-moment correlation scores between samples were calculated using binary matrices representing the status of most frequently mutated genes (1 = mutant, 0 = wild-type), yielding a matrix of quantified genomic correlation. These data were subject to hierarchical clustering using Euclidean distance and Ward’s linkage. Bars denote mutation in *CTNNB1* and *TP53*.

**Fig. 3:**
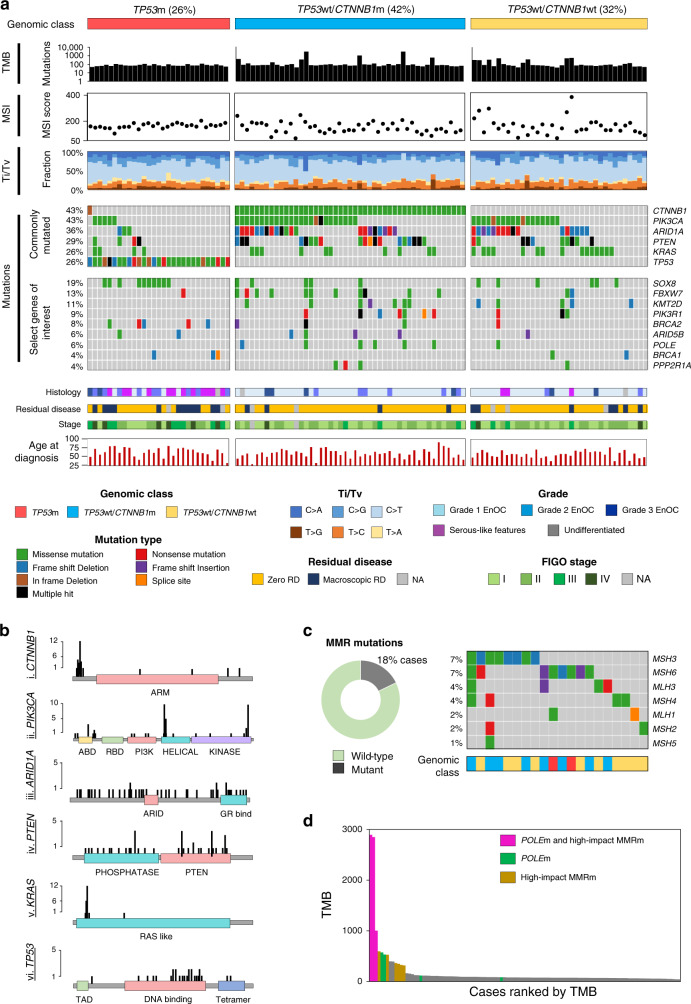
Genomic characterisation of endometrioid ovarian carcinoma (EnOC). **a** Whole exome sequencing identifies three major genomic subtypes of endometrioid ovarian carcinoma based on *TP53* and *CTNNB1* mutation status. Molecular signatures for each tumour are plotted as the fraction of transversions and transitions. Mutations are displayed as an oncoplot. Grey denotes no mutation. Upper plot shows the six most frequently mutated genes; lower plot shows select genes of interest. **b** Lollipop plots of five common gene targets of mutation. **c** Mismatch repair (MMR) mutations across cases. **d** Relationship between tumour mutational burden (TMB) and mutations in *POLE* and/or MMR genes. Ti transition, tv transversion, MSI microsatellite instability, *RD* residual disease.

The most commonly mutated genes included *CTNNB1* (48 cases, 42.9%), *PIK3CA* (48 cases, 42.9%), *ARID1A* (40 cases, 35.7%), *PTEN* (33 cases, 29.5%), *KRAS* (29 cases, 25.9%), and *TP53* (29 cases, 25.9%) (Supplementary Fig. 1). Unsupervised hierarchical clustering across the 50 most commonly mutated genes revealed mutation of *TP53* and *CTNNB1* (*TP53*m and *CTNNB1*m) as the most prominent stratifying events (Fig. 2 and Supplementary Fig. 2). *TP53*m and *CTNNB1*m were largely mutually exclusive (Fig. 3a), with significant depletion of *CTNNB1*m in the *TP53*m group (*P* < 0.001; co-occurrence in one case, 0.9%). The *CTNNB1*m rate in the *TP53* wild-type (*TP53*wt) group was 56.6% (47 of 83 *TP53*wt cases).

Mutation of *SOX8*, a gene target of mutational disruption previously unreported at high frequency in EnOC, was also identified as a common event (21 cases, 18.8%), alongside other targets of mutation (Fig. 3a and Supplementary Figs. 1, 3A). There was significant enrichment of *SOX8*m in the *TP53*m group (10/29, 34.5% *SOX8*m in the *TP53*m group vs. 11/83, 13.3% *SOX8*m in the *TP53*wt group; *P* = 0.025). Events in other genes previously reported as mutated in EnOC or endometrial cancer were identified at lower frequency, including *FBXW7*m (14 cases, 12.5%), *KMT2D/MLL3*m (12 cases, 10.7%)*, BRCA1/2*m (14 cases, 12.5%) (Supplementary Table 1), *PIK3R1*m (10 cases, 8.9%), *MTOR*m (7 cases, 6.3%), *POLE*m (7 cases, 6.3%) (Supplementary Fig. 3B), *APC* (6 cases, 5.4%) and *PPP2R1A*m (4 cases, 3.6%) (Fig. 3a and Supplementary Fig. 1). *BRCA1*/*2*m cases demonstrated a high rate of *TP53*m (57.1%, 8 of 14 cases) and a high rate of mutation in genes previously reported in EnOC (71.4%, 10 of 14 cases with mutations in *ARID1A*, *CTNNB1*, *PTEN*, *PIK3CA*, *KRAS*, or genes encoding mismatch repair proteins).

Pathway analysis identified a large number of mutations across four major oncogenic pathways: PI3K-AKT, WNT, RAS, and NOTCH (Supplementary Fig. 4).

### Tumour mutational burden (TMB) and microsatellite instability (MSI)

A median of 78 variants were detected per sample (range 42–2894) (Supplementary Figs. 5, 6A). Ten cases (8.9%) were considered hypermutated (>250 mutations per sample) and 3 (2.7%) were considered ultramutated (>1000 mutations) (Fig. 3a). Overall analysis of TMB against TCGA derived datasets places EnOC alongside HGSOC (median TMB = 72), colonic adenocarcinoma (median TMB = 76) and EnEC (median TMB = 78) (Supplementary Fig. 6B).

Mutations in one or more genes encoding MMR proteins were identified in 20 cases (17.9%) (Fig. 3c), most commonly in *MSH3, MSH6*, *MLH3* or *MSH4*. The majority of MMR-mutant (MMRm) tumours were *TP53*wt (18/20 cases, 90.0%). High impact MMRm (frameshifting InDels, nonsense or splice site mutations) were associated with significantly higher MSI scores compared to those containing missense MMRm (median 236 vs. 142.5, *P* < 0.001) and MMRwt samples (median 236 vs. 162, *P* < 0.001) (Fig. 3a and Supplementary Fig. 7). There was no significant difference in MSI score between MMRwt cases and those with missense MMRm (*P* = 0.194).

*POLE*m commonly occured over a hotspot within the exonuclease domain (42.9%, 3 of 7 *POLE*m cases) (Supplementary Fig. 3B). There was a high frequency of concurrent *POLE*m and MMRm (five of seven *POLE*m cases, 71.4%), with significant enrichment for MMRm within the *POLE*m versus *POLE*wt group (71.4%, 5/7 vs. 14.3%, 15/105, *P* = 0.002). Together, the *POLE*m and high impact MMRm cases accounted for the majority of high TMB cases (Fig. 3d). Cases with concurrent *POLE*m and MMRm accounted for all three ultramutated tumours; 8 of the 10 (80.0%) hypermutated tumours contained either *POLE*m or high impact MMRm.

### Mutational spectrum in endometrioid ovarian carcinoma

We observed a bias towards C>T and C>A transversion and transition molecular signatures across the 112 EnOC cases (Fig. 3a). A shift in signatures was observed in samples harbouring *POLE*m, with a greater proportion of T>G changes, and depletion of C>G and T>A substitutions in this population (Supplementary Fig. 8).

### Tumour genomic complexity and copy number alterations

**Fig. 4:**
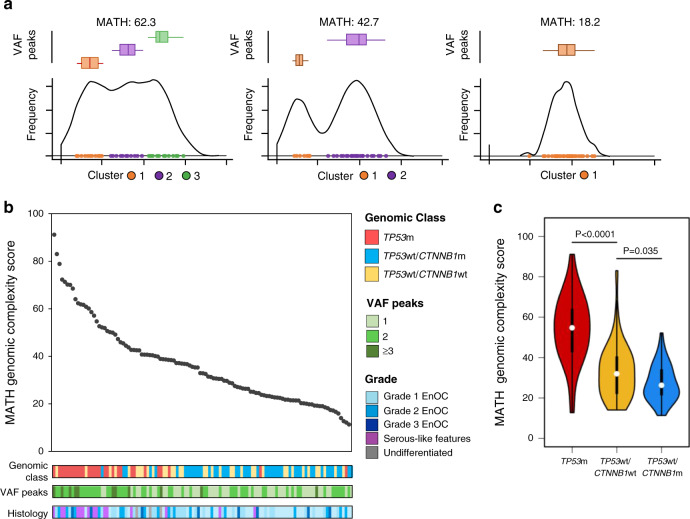
Genomic complexity of endometrioid ovarian carcinomas (EnOC). **a** Representative examples of variant allele frequency density plots for a high complexity tumour (left), intermediate complexity tumour (centre) and low complexity tumour (right). Variant allele frequency (VAF) clusters are shown and summarised within box plots above the density plot; boxes represent the 1st to 3rd quartile, with the median labelled as the central line, and whiskers extend to the data range from 1st and 3rd quartile +/−1.5 times the interquartile range. **b** Mutant-allele tumour heterogeneity (MATH) genomic complexity scores across all 112 endometrioid ovarian carcinoma (EnOC) cases, ranked high to low. **c** MATH genomic complexity scores across the three EnOC subtypes. Comparisons were made using the two-sided Mann–Whitney *U*-test without adjustment for multiple testing (*P* < 0.0001 and *P* = 0.0353). m mutant, wt wild-type.

Distribution of per-sample global variant allele frequency (VAF) density and calculation of mutant-allele tumour heterogeneity (MATH) score across the 112 EnOC cases was used to infer tumour genomic complexity (Fig. 4). *TP53*wt tumours were predominantly low complexity, demonstrating lower MATH scores (median 27.5 vs. 54.7, *P* < 0.001) (Fig. 4b, c and Supplementary Fig. 9A) and fewer discrete VAF peaks (*P* < 0.001) (Supplementary Table 2) compared to *TP53*m cases.

Analysis of CNA events across samples revealed differential CNA burden across molecular subgroups defined by *TP53*m and *CTNNB1*m status, with *TP53*m cases harbouring greater CNA burden compared to *TP53*wt tumours (*P* < 0.0001) (Supplementary Figs. 9B, 10A, B). The most frequent CNA events across the cohort were gain of *ZNF43* (30 cases, 26.8%), *RABA1C* (24 cases, 21.4%) and *AMY1C* (23 cases, 20.5%), and loss of *PKNOX1* (42 cases, 37.5%), *CEP68* (29 cases, 25.9%) and *GLB1L* (20 cases, 17.9%) (Supplementary Fig. 10C). CNA events were also identified over genes that were frequent targets of mutational disruption (Supplementary Fig. 10D).

### Molecular events define clinically distinct disease subtypes

*TP53*m cases demonstrated significantly inferior DSS upon univariable analysis (HR = 4.43, 95% CI 2.27–8.64, Bonferroni-adjusted *P* < 0.001) (Supplementary Figs. 11A, 12A and Supplementary Table 3), were more likely to be diagnosed at advanced stage (14 of 29 evaluable cases, 48.3% stage III/IV vs. 10 of 81, 12.3%; *P* < 0.001) (Supplementary Table 4), less likely to be successfully resected to zero macroscopic residual disease (RD) (44.4%, 12 of 27 evaluable cases with macroscopic RD after surgical debulking vs. 14.1%, 11 of 78; *P* = 0.003), and demonstrated a trend for greater age at diagnosis which did not meet statistical significance (median 61 vs. 57 years, *P* = 0.063). Multivariable analysis accounting for patient age, stage at diagnosis, and extent of RD following primary cytoreduction identified *TP53*m as independently associated with shorter DSS (*P* = 0.031) (Supplementary Tables 5, 6). The *TP53*m group demonstrated significant depletion of cases with concurrent endometrial cancer diagnosis (3.4%, 1 of 29 *TP53*m vs. 21.7%, 18 of 83 *TP53*wt, *P* = 0.023). The median DSS and PFS for *TP53*m cases were 3.78 and 1.54 years, respectively.

**Fig. 5:**
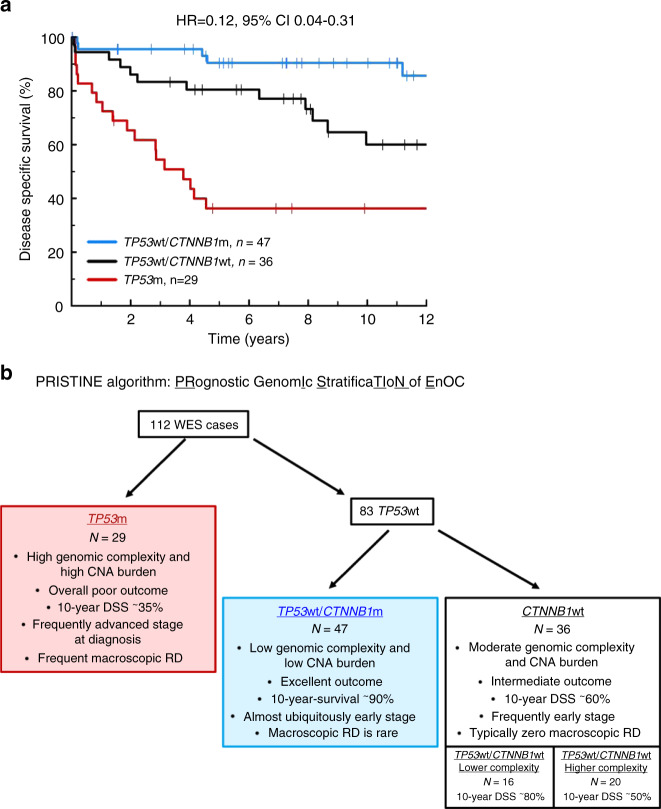
Genomic subtypes of endometrioid ovarian carcinoma demonstrate distinct clinical behaviour. **a** Disease-specific survival based on *TP53*m and *CTNNB1*m status; labelled hazard ratio represents comparison of the *TP53*wt/*CTNNB1*m group with the *TP53*m group. HR for *TP53*wt/*CTNNB1*wt vs. *TP53*m = 0.37, 95% CI 0.18–78; HR for *TP53*wt/*CTNNB1*m vs. *TP53*wt/*CTNNB1*wt = 0.31, 95% CI 0.11–0.88. **b** Summary of the PRISTINE algorithm for molecular subtyping in endometrioid ovarian carcinoma. m mutant, wt wild-type, RD residual disease, WES whole exome sequencing, DSS disease-specific survival.

By contrast, cases with *CTNNB1*m were overwhelmingly stage I/II at diagnosis (89.1%, 41 of 46 evaluable cases) and debulked to zero macroscopic RD (87.0%, 42 of 46 evaluable cases), with markedly favourable outcome (HR for DSS = 0.23, 95% CI 0.10–0.56, Bonferroni-adjusted *P* = 0.010) (Supplementary Figs. 11B, 12B and Supplementary Table 3) which was significant upon multivariable analysis (*P* = 0.017) (Supplementary Tables 7, 8). *CTNNB1*m was significantly associated with favourable outcome specifically in the context of *TP53*wt cases (HR for DSS = 0.31, 95% CI 0.11–0.88) (Fig. 5a), and *TP53*wt/*CTNNB1*m cases were less genomically complex vs. their *TP53*wt/*CTNNB1*wt counterparts (median MATH score 26.3 vs. 31.9, *P* = 0.035) with fewer CNA events (*P* = 0.042) (Fig. 4c and Supplementary Fig. 10B). The DSS difference between genomic subgroups defined by combined *TP53*m and *CTNNB1*m status was maintained upon exclusion of cases with concurrent endometrial carcinoma (*P* < 0.001) (Supplementary Fig. 13) and upon exclusion of cases demonstrating *TP53*m in the absence of endometrioid-associated mutations (*P* < 0.001) (Supplementary Fig. 14).

*POLE*m EnOC cases did not demonstrate an obvious exceptional 5-year survival pattern akin to that reported in *POLE*m endometrial carcinomas, and did not demonstrate prolonged DSS vs. *POLE*wt patients (5-year DSS 85.7%; *P* = 0.337 vs. *POLE*wt) (Supplementary Table 3), although case numbers were extremely low (*n* = 7).

Greater tumour complexity was associated with inferior survival when defined by the number of VAF peaks (*P* = 0.020 for DSS) or continuous MATH score (*P* < 0.001 for DSS) (Supplementary Table 9). Exploratory analysis to determine whether the outcome of *TP53*wt/*CTNNB1*wt cases could be further resolved by genomic complexity identified that low complexity *TP53*wt/*CTNNB1*wt cases (MATH score ≤ median MATH score in EnOCs with a single VAF peak) demonstrated relatively favourable outcome (10-year DSS 77.0%) (Supplementary Fig. 15), while their more genomically complex counterparts demonstrated intermediate prognosis (10-year DSS 48.7%); however, this difference did not reach statistical significance and case numbers were limited.

Together, these data support a clinically meaningful classification system driven *TP53*m and *CTNNB1*m status in a step-wise fashion, which we present here as the PRISTINE algorithm: PRognostic genomIc StratificaTIoN of EnOC (Fig. 5b). Genomic complexity may represent a potential tool to further discriminate outcome in these subtypes.

### Utility of immunohistochemistry to identify disease subtypes

IHC for p53 and β-catenin, the protein products of *TP53* and *CTNNB1*, was able to identify subgroups of EnOC with differential DSS (*P* < 0.001 and *P* = 0.045) (Supplementary Fig. 16A, B). Combined use of these markers to recapitulate the PRISTINE algorithm identified patient groups with differential DSS akin to those defined by genomic data (*P* < 0.001) (Supplementary Fig. 16C). However, aberrant β-catenin expression (βcat-aberrant) resolved outcome less well in those with wild-type p53 protein expression (p53-wt) compared to *CTNNB1*m status in the *TP53*wt group (Fig. 5a), likely owing to the suboptimal sensitivity of β-catenin IHC for *CTNNB1*m (Supplementary Fig. 16B).

## Discussion

The molecular landscape of EnOC is poorly defined, particularly in high grade cases, due to under-investigation and historic misclassification of HGSOC as high grade EnOC in older studies. WT1 negativity has emerged as an important discriminator of high grade EnOC from HGSOC, which displays morphological similarities^4,24–27^. To our knowledge, this is the largest report of genomically characterised EnOC to date, utilising routine WT1 IHC to exclude pseudo-endometrioid HGSOC that have contaminated previous studies of this cancer type.

In line with previous sequencing studies in small cohorts of EnOC, we identified a high mutation rate of genes known to be perturbed in EnEC, the most frequent of which were *CTNNB1*, *PIK3CA*, *PTEN*, *ARID1A*, *KRAS*, and *TP53*^8,11,22,28,29^. Our EnOC cohort demonstrated a similar rate of *TP53*m to the TCGA study of EnEC^11^; by contrast, the mutation rate of *PTEN* was lower. We demonstrate that EnOC tumours contain a moderate TMB with respect to other cancer types, with a small proportion hyper-mutated or ultra-mutated in nature (11.6%).

MMR deficiency due to mutations or methylation in MMR protein-encoding genes results in MSI, and loss of MMR protein expression has previously been demonstrated in approximately 7–14% of EnOC^30–34^. As in EnEC, we identify a subgroup of EnOC harbouring MMRm, with a similar overall MMRm rate as reported previously in EnOC^30–34^. We demonstrate that cases with high impact MMRm (nonsense, frameshifting or splice site mutations) demonstrate MSI and that these cases account for many of the samples demonstrating high TMB. *POLE*m was rare in this cohort (6.3% of cases); there was significant enrichment for MMRm within this population (*P* = 0.002) and concurrent mutation of *POLE* and MMR genes was present in all three ultramutated tumours. Notably, when considering only missense MMRm, we observed no significant difference in MSI score compared to MMRwt cases, suggesting fewer missense mutations lead to functional loss of MMR. Moreover, *POLE*m cases did not appear to demonstrate the exceptional 5-year survival reported in *POLE*m endometrial carcinomas^11^, and was not associated with significantly prolonged survival, though the power of this analysis was severely limited. The low *POLE*m rate we observed is consistent with previous reports in EnOC^35,36^.

Collectively, the data presented here identify *TP53*m EnOC as a distinct clinical and biological subtype of disease. *TP53*m cases demonstrated higher levels of CNA events, greater tumour genomic complexity, higher rate of advanced stage at diagnosis, inferior rate of complete macroscopic tumour resection, and overall poor clinical outcome. This is consistent with the poor prognosis reported in EnEC harbouring *TP53*m^11^, is reminiscent of HGSOC^12^, and is in line with several studies of EnOC^28,37^. In particular, the study performed by Parra-Herran et al.^28^ applied the PROMISE algorithm, an EnEC molecular classifier, to a cohort of WT1 negative EnOC and found the p53-aberrant group to have the worst survival; a recent study of early stage EnOC also reported poor outcome for cases with p53-aberrant cases^38^. In our cohort, *TP53*m cases also represented those least likely to demonstrate concurrent endometrial cancer.

While the copy number and survival profile of our *TP53*m EnOC group provides one rationale for reclassification of these tumours as HGSOC, the high frequency (48.3%, 14 of 29) of classic EnOC mutations (*CTNNB1*, *PTEN*, *ARID1A*, *KRAS, PIK3CA*, or MMRm), lack of WT1 expression (in all cases) and high rate of early stage diagnosis in this cohort (51.7%, 15 stage I/II) form a compelling argument that these represent true EnOC. Indeed, these poorer prognosis EnOC cases may well be suitable for inclusion alongside true HGSOC in trials of novel therapeutic strategies for aggressive OC types. Only nine cases (8.0% of our cohort) represented feasible candidates as possible true WT1-negative HGSOC (advanced stage at diagnosis and *TP53*m without mutations suggestive of endometrioid carcinoma i.e., MMR, *PTEN*, *CTNNB1*, *PIK3CA*, *ARID1A* and *KRAS* wild-type), which are recognised as a rare phenomenon (≤5% HGSOC cases)^24^. The true histological subtype of this small group cannot be determined with absolute certainty; however, sensitivity analysis excluding possible occult serous carcinomas of ovarian or endometrial origin identified the same outcome differences between genomic subtypes.

Conversely, *CTNNB1*m—which appears mutually exclusive with *TP53*m—is associated with early-stage disease of low genomic complexity that is easily debulked to zero macroscopic RD, and these cases demonstrate excellent clinical outcome. This is in contrast to findings in EnEC associating *CTNNB1*m with a greater chance of recurrence^39^. Within our EnOC cohort, *CTNNB1*m status was also associated with favourable outcome specifically in the context of *TP53*wt tumours, suggesting clinical impact independent of its anti-correlation with *TP53*m. These data support the notion of a tiered classification system driven by *TP53*m and *CTNNB1*m status to define molecular subtypes of EnOC with markedly differential clinical outcome and clinicopathological features; this classifier—the PRISTINE algorithm—should now be validated in an independently curated, sufficiently powered EnOC dataset defined using contemporary criteria, including the use of WT1 IHC. We demonstrate that consideration of genomic complexity may provide a potential future way to further resolve outcomes within these subgroups, though this analysis was exploratory in nature.

Despite the variability in processing, age, fixation and preservation of tissue specimens, we demonstrate that IHC for p53 and β-catenin proteins can recapitulate this classification system, but is limited by the sensitivity of β-catenin IHC for detecting *CTNNB1*m, consistent with previous reports of β-catenin immunostaining as a surrogate for *CTNNB1*m^40,41^. Implementation of IHC-based classification may well demonstrate improved sensitivity/specificity in newly diagnosed cases with uniformly fixed, well preserved whole-slide tissue sections.

The high rate of genomic disruption in *CTNNB1*, *KRAS*, *PTEN* and *PIK3CA* suggests that inhibitors of the WNT, MAPK and PI3K pathways represent agents with potential clinical utility in EnOC treatment. Efforts to identify novel therapeutic strategies should focus on cases with greatest unmet clinical need, namely *CTNNB1*wt cases. In particular, *TP53*m cases represent those where further treatment options are urgently required to improve outcome, and we identified potentially clinically actionable mutations in a large proportion of these cases (14% with *KRAS*m, 28% with *PTEN*m/*PIK3CA*m, 28% with *BRCA1/2*m).

Finally, we identify *SOX8* as a gene target of recurrent mutation in EnOC. SRY-related high mobility group box (*SOX*) genes encode a family of transcription factors, which act as critical regulators of cellular programming and are frequently altered in many cancers^42^. Interestingly, analysis of TCGA data reveals that *SOX8*m occurs at low frequency in uterine cancers (3% in uterine corpus endometrial carcinoma, 1% in uterine carcinosarcoma) as well as in colonic adenocarcinoma (2%) (data from the TCGA portal^43^). As recent studies suggest that *SOX8* in part regulates the activity of genes associated with the WNT/β-catenin pathway, a commonly mutated pathway in EnOC, mutation of this gene may impact on classically defined EnOC pathways through this route^44^. Genomic disruption of *SOX8* therefore represents a previously unreported candidate mechanism by which EnOC may frequently perturb the WNT/β-catenin pathway. Given the frequent co-occurrence of *SOX8*m and *TP53*m in our cohort (34.5% *SOX8*m in the *TP53*m group), selection bias against true high grade EnOC in previous studies—leading to depletion of *TP53*m cases in those cohorts—may explain why *SOX8*m has not previously been identified as a common genomic event in EnOC.

Beyond genomic characterisation, a transcriptomic study of EnOC identified clinically meaningful patient subgroups defined at the gene expression level akin to those previously described in HGSOC^18^. Interestingly, while the poorer prognosis of genomically complex EnOC cases we describe here might be expected to produce a more pronounced anti-tumour immune response, the immunoreactive transcriptomic subgroup they identified did not demonstrate the poorest outcome. Future work should seek to correlate EnOC subgroups defined at these different levels in order to determine the association between genomic and transcriptomic events in this tumour type. Similarly, comparison with subgroups defined at the proteomic level, including those based on expression patterns of hormone receptors^45^, should be made.

In summary, we have demonstrated that EnOC is a molecularly heterogeneous disease, comprising multiple genomic subtypes. These subtypes demonstrate differential clinical outcome and clinicopathological features. In particular, our study highlights *CTNNB1*m and *TP53*m as markers of biologically distinct subtypes of EnOC with contrasting clinical behaviour. These markers have the potential to inform future prognostication and molecular stratification within EnOC. Gene sequencing of *TP53* and *CTNNB1*, or IHC directed at their respective gene products, represent mechanisms by which these findings could readily be translated into routine risk-stratification of newly diagnosed cases. Patients with EnOC demonstrating absence of *CTNNB1*m and/or presence of *TP53*m have the greatest unmet clinical need; many of these tumours harbour activating mutations in pathways that may be targetable with molecular agents. Investigating the clinical efficacy of inhibitors of the MAPK/RAS, WNT and PI3K pathways has the potential to identify agents that will improve EnOC patient survival.

## Methods

### Ethical approval

Ethical approval for the use of human tissue specimens for research was obtained from South East Scotland Scottish Academic Health Sciences Collaboration BioResource (reference 15/ES/0094-SR494). Correlation of molecular data to clinical outcome and clinicopathological variables in ovarian cancer was approved by NHS Lothian Research and Development (reference 2007/W/ON/29). All relevant ethical regulations have been complied with, including the need for written informed consent where required.

### Pathology review and immunohistochemistry

505 patients diagnosed with OC between August 1968 and May 2014, and whose pathology reports contained the term “endometrioid”, were identified through the Edinburgh Ovarian Cancer Database (Fig. 1); tumour material was available for 289 cases^45^. Chemotherapy naïve tumour from the primary site was selected where available. Pathology review was conducted as per WHO 2014 classification, including IHC for WT1 in every case (see Supplementary Methods), by an expert gynaecological pathologist (CSH). A confirmatory observer (BS) was present for all pathology review. The presence of endometriosis was recorded from the reviewed slides or pathology report.

Cases with non-interpretable morphology, non-evaluable tumour and cases representing metastases from primary endometrial cancer, as defined by WHO criteria, were excluded. Ovarian metastases, WT1 positive tumours, carcinosarcomas and carcinomas of clear cell, mucinous or mixed histology were also excluded (Fig. 1). IHC for cytokeratin 7 and cytokeratin 20 (CK7 and CK20) was performed to exclude colorectal adenocarcinoma metastases (see Supplementary Methods). p53 and β-catenin IHC was performed as described in the Supplementary Methods.

### Clinical data

Baseline characteristics and outcome data were extracted from the Edinburgh Ovarian Cancer Database, wherein the diagnostic, treatment and follow-up data for every ovarian cancer patient treated at the Edinburgh Cancer Centre is prospectively entered as a part of routine care^5^. DSS was calculated from the date of pathologically confirmed OC diagnosis. PFS was recorded as the duration between the date of diagnosis to the date of first radiological progression or recurrence, or death from EnOC.

### DNA extraction

H&E-stained slides were marked by an expert gynaecological pathologist (CSH) to identify tumour areas suitable for macrodissection in order to enrich for tumour cellularity. DNA extraction was performed using the QIAamp DNA formalin-fixed paraffin-embedded (FFPE) Tissue Kit (Qiagen, Venlo, Netherlands) and Deparaffinization Solution according to the manufacturer’s instructions.

### Whole exome sequencing

Exome capture was performed using the Illumina TruSeq Exome Library Prep kit (see Supplementary Methods) and WES was performed on the Illumina NextSeq 550 (Illumina, Inc., San Diego, CA, USA). The median per-sample on-target coverage in the successfully sequenced samples was 89.5× (range 36×–289×). Data were aligned to the GRCh38 human reference genome using bwa-0.7.17^46^, duplicates marked and base quality scores recalibrated with the GenomeAnalysisToolkit (GATK) v4^47^ in the bcbio pipeline (see Supplementary Methods).

### Variant calling and classification

Variant calling was performed using a majority vote system from three variant caller algorithms: VarDict^48^, Mutect2^49^ and Freebayes^50^. Filtering for FFPE and oxidation artifacts was applied using GATK CollectSequencingArtifactMetrics and FilterByOrientationBias. Variants associated with low sequence depth (<20×) or low variant allele frequency (<10%) were removed. Common variants were excluded using the 1000 genomes and ExAC reference datasets; known pathogenic and benign variants were flagged using ClinVar^51^, and remaining variants were filtered to remove likely non-functional variation using the Polymorphism Phenotyping (PolyPhen)^52^ and Sorting Intolerant from Tolerant^53^ functional prediction tools (see Supplementary Methods).

MSI score was assessed as the number of short insertions or deletions (InDels) detected in a given sample. TMB was defined as the number of mutations present in a given tumour following filtering. TMB across other cancer datasets in The Cancer Genome Atlas (TCGA) were contrasted against those in our EnOC datasets^54^. Transitions and transversions were calculated using the titv function in maftools^55^.

Unsupervised analysis was performed using the top 50 most frequently mutated genes represented as a binary matrix (0, wild-type; 1, mutant). Product-moment correlation scores were calculated between these binary signatures of each sample to form a matrix of quantified genomic correlation. Samples were then clustered by Euclidian distance and Ward’s linkage based on this correlation matrix. Heat maps were drawn in R using the ggplot package. Supervised mutational analysis was performed using the most commonly mutated genes across sequenced samples. Genomic events in these genes and overall TMB analysis were visualised using the R package maftools^55^. Pathway analysis was carried out using the OncogenicPathways function^56^.

### Tumour genomic complexity scoring

Tumour genomic complexity was assessed by VAF density using the inferHeterogeneity function in the R package maftools^55,57^ (see Supplementary Methods). Resulting MATH scores represent the width of the VAF distribution; specimens of low complexity with a single driver event and associated outgrowth demonstrate fewer VAF peaks with a lower MATH score. Conversely, highly complex tumours with multiple driver events, branched evolution and multiple subclonal populations demonstrate multiple VAF peaks and higher MATH score.

### Copy number alteration detection

Copy number analysis was performed using GeneCN pipelines in Bio-DB-HTS version 2.10 to identify regions of significant copy number gain or loss (copy number score >5 standard deviations from reference, *P* < 0.05) using the pooled *TP53*wt samples as a reference population.

### Statistical analysis

Statistical analyses were performed using R version 4.0.0. Comparisons of continuous data were made with the Mann–Whitney *U*-test or *T*-test, as appropriate. Median follow-up time was calculated using the reverse Kaplan–Meier method. Survival analysis was performed using Cox proportional hazards regression models in the Survival package. Multivariable analyses accounted for FIGO stage, patient age at diagnosis, decade of patient diagnosis and extent of RD following surgical cytoreduction. Comparisons of frequency were performed using the Chi-square test or Fisher’s exact test, as appropriate. Correction for multiplicity of testing was performed using the Bonferroni method where appropriate.

### Reporting summary

Further information on research design is available in the Nature Research Reporting Summary linked to this article.

## Supplementary information

Supplementary Information

Reporting Summary

## Data Availability

The primary and processed data used to generate the analyses presented here are available via the European Genome-phenome Archive (accession EGAS00001004366) upon request to our data access committee; committee approval is required to comply with the local research ethics framework. For more information please see [https://ega-archive.org/access/data-access]. The remaining data are available in the Article, Supplementary Information or available from the authors upon request. The 1000 Genomes and ExAC reference datasets can be found at [http://www.internationalgenome.org] (version: phase 1 SNP and InDel) and [http://exac.broadinstitute.org] (version ExAC.0.3.GRCh38).

## References

[1] Vaughan S (2011). Rethinking ovarian cancer: recommendations for improving outcomes. Nat. Rev. Cancer.

[2] Hollis RL, Gourley C (2016). Genetic and molecular changes in ovarian cancer. Cancer Biol. Med..

[3] Storey DJ (2008). Endometrioid epithelial ovarian cancer: 20 years of prospectively collected data from a single center. Cancer.

[4] Lim D (2016). Morphological and immunohistochemical reevaluation of tumors initially diagnosed as ovarian endometrioid carcinoma with emphasis on high-grade tumors. Am. J. Surg. Pathol..

[5] Irodi, A. et al. Patterns of clinicopathological features and outcome in epithelial ovarian cancer patients: 35 years of prospectively collected data. *BJOG* **127**, 1409–1420 (2020). 10.1111/1471-0528.16264 32285600

[6] Moore K (2018). Maintenance olaparib in patients with newly diagnosed advanced ovarian cancer. N. Engl. J. Med..

[7] Mirza MR (2016). Niraparib maintenance therapy in platinum-sensitive, recurrent ovarian cancer. N. Engl. J. Med..

[8] Huang HN (2015). Ovarian and endometrial endometrioid adenocarcinomas have distinct profiles of microsatellite instability, PTEN expression, and ARID1A expression. Histopathology.

[9] Geyer JT (2009). Pathogenetic pathways in ovarian endometrioid adenocarcinoma: a molecular study of 29 cases. Am. J. Surg. Pathol..

[10] Wu R (2007). Mouse model of human ovarian endometrioid adenocarcinoma based on somatic defects in the Wnt/beta-catenin and PI3K/Pten signaling pathways. Cancer Cell.

[11] Kandoth C (2013). Integrated genomic characterization of endometrial carcinoma. Nature.

[12] Bell D (2011). Integrated genomic analyses of ovarian carcinoma. Nature.

[13] Cybulska P (2019). Molecular profiling and molecular classification of endometrioid ovarian carcinomas. Gynecol. Oncol..

[14] Kurman R. J., Carcangiu M. L., Herrington C. S., Young R. H. (Eds). *WHO Classification of Tumours of Female Reproductive Organs* (WHO Press, Hoboken, 2014).

[15] Soslow RA (2012). Morphologic patterns associated with BRCA1 and BRCA2 genotype in ovarian carcinoma. Mod. Pathol..

[16] Madore J (2010). Characterization of the molecular differences between ovarian endometrioid carcinoma and ovarian serous carcinoma. J. Pathol..

[17] Schwartz DR (2002). Gene expression in ovarian cancer reflects both morphology and biological behavior, distinguishing clear cell from other poor-prognosis ovarian carcinomas. Cancer Res..

[18] Winterhoff B (2016). Molecular classification of high grade endometrioid and clear cell ovarian cancer using TCGA gene expression signatures. Gynecol. Oncol..

[19] Tothill RW (2008). Novel molecular subtypes of serous and endometrioid ovarian cancer linked to clinical outcome. Clin. Cancer Res..

[20] Assem H (2018). High-grade endometrioid carcinoma of the ovary: a clinicopathologic study of 30 cases. Am. J. Surg. Pathol..

[21] Soyama H (2018). A pathological study using 2014 WHO criteria reveals poor prognosis of grade 3 ovarian endometrioid carcinomas. Vivo.

[22] McConechy MK (2014). Ovarian and endometrial endometrioid carcinomas have distinct CTNNB1 and PTEN mutation profiles. Mod. Pathol..

[23] Wang YK (2017). Genomic consequences of aberrant DNA repair mechanisms stratify ovarian cancer histotypes. Nat. Genet..

[24] Kobel M (2016). An immunohistochemical algorithm for ovarian carcinoma typing. Int. J. Gynecol. Pathol..

[25] Acs G, Pasha T, Zhang PJ (2004). WT1 is differentially expressed in serous, endometrioid, clear cell, and mucinous carcinomas of the peritoneum, fallopian tube, ovary, and endometrium. Int. J. Gynecol. Pathol..

[26] Cathro HP, Stoler MH (2005). The utility of calretinin, inhibin, and WT1 immunohistochemical staining in the differential diagnosis of ovarian tumors. Hum. Pathol..

[27] Al-Hussaini M, Stockman A, Foster H, McCluggage WG (2004). WT-1 assists in distinguishing ovarian from uterine serous carcinoma and in distinguishing between serous and endometrioid ovarian carcinoma. Histopathology.

[28] Parra-Herran C (2017). Molecular-based classification algorithm for endometrial carcinoma categorizes ovarian endometrioid carcinoma into prognostically significant groups. Mod. Pathol..

[29] Stewart CJ (2012). KRAS mutations in ovarian low-grade endometrioid adenocarcinoma: association with concurrent endometriosis. Hum. Pathol..

[30] Lu FI (2012). Prevalence of loss of expression of DNA mismatch repair proteins in primary epithelial ovarian tumors. Int. J. Gynecol. Pathol..

[31] Aysal A (2012). Ovarian endometrioid adenocarcinoma: incidence and clinical significance of the morphologic and immunohistochemical markers of mismatch repair protein defects and tumor microsatellite instability. Am. J. Surg. Pathol..

[32] Liu J (2004). Microsatellite instability and expression of hMLH1 and hMSH2 proteins in ovarian endometrioid cancer. Mod. Pathol..

[33] Bennett JA (2019). Incidence of mismatch repair protein deficiency and associated clinicopathologic features in a cohort of 104 ovarian endometrioid carcinomas. Am. J. Surg. Pathol..

[34] Rambau PF (2016). Significant frequency of MSH2/MSH6 abnormality in ovarian endometrioid carcinoma supports histotype-specific Lynch syndrome screening in ovarian carcinomas. Histopathology.

[35] Hoang LN (2015). Polymerase epsilon exonuclease domain mutations in ovarian endometrioid carcinoma. Int. J. Gynecol. Cancer.

[36] Zou Y (2014). Frequent POLE1 p.S297F mutation in Chinese patients with ovarian endometrioid carcinoma. Mutat. Res..

[37] Okuda T (2003). p53 mutations and overexpression affect prognosis of ovarian endometrioid cancer but not clear cell cancer. Gynecol. Oncol..

[38] Leskela S (2020). Molecular heterogeneity of endometrioid ovarian carcinoma: an analysis of 166 cases using the endometrial cancer subrogate molecular classification. Am. J. Surg. Pathol..

[39] Kurnit KC (2017). CTNNB1 (beta-catenin) mutation identifies low grade, early stage endometrial cancer patients at increased risk of recurrence. Mod. Pathol..

[40] Kim G (2018). Nuclear β-catenin localization and mutation of the CTNNB1 gene: a context-dependent association. Mod. Pathol..

[41] Travaglino A (2019). Immunohistochemical nuclear expression of β-catenin as a surrogate of CTNNB1 exon 3 mutation in endometrial cancer. Am. J. Clin. Pathol..

[42] Thu KL (2014). SOX15 and other SOX family members are important mediators of tumorigenesis in multiple cancer types. Oncoscience.

[43] Cerami E (2012). The cBio cancer genomics portal: an open platform for exploring multidimensional cancer genomics data. Cancer Discov..

[44] Xie SL (2018). SOX8 regulates cancer stem-like properties and cisplatin-induced EMT in tongue squamous cell carcinoma by acting on the Wnt/beta-catenin pathway. Int. J. Cancer.

[45] Hollis RL (2019). Hormone receptor expression patterns define clinically meaningful subgroups of endometrioid ovarian carcinoma. Gynecol. Oncol..

[46] Li H, Durbin R (2010). Fast and accurate long-read alignment with Burrows-Wheeler transform. Bioinformatics.

[47] McKenna A (2010). The Genome Analysis Toolkit: a MapReduce framework for analyzing next-generation DNA sequencing data. Genome Res..

[48] Lai Z (2016). VarDict: a novel and versatile variant caller for next-generation sequencing in cancer research. Nucleic Acids Res..

[49] Cibulskis K (2013). Sensitive detection of somatic point mutations in impure and heterogeneous cancer samples. Nat. Biotechnol..

[50] Garrison, E. & Marth, G. Haplotype-based variant detection from short-read sequencing. Preprint at https://arxiv.org/abs/1207.3907 (2012).

[51] Landrum MJ (2014). ClinVar: public archive of relationships among sequence variation and human phenotype. Nucleic Acids Res..

[52] Adzhubei IA (2010). A method and server for predicting damaging missense mutations. Nat. Methods.

[53] Ng PC, Henikoff S (2003). SIFT: predicting amino acid changes that affect protein function. Nucleic Acids Res..

[54] Alexandrov LB (2013). Signatures of mutational processes in human cancer. Nature.

[55] Mayakonda A, Lin DC, Assenov Y, Plass C, Koeffler HP (2018). Maftools: efficient and comprehensive analysis of somatic variants in cancer. Genome Res..

[56] Sanchez-Vega F (2018). Oncogenic signaling pathways in The Cancer Genome Atlas. Cell.

[57] Mroz EA, Rocco JW (2013). MATH, a novel measure of intratumor genetic heterogeneity, is high in poor-outcome classes of head and neck squamous cell carcinoma. Oral. Oncol..

