# Supplementary material for: Molecular stratification of endometrioid ovarian carcinoma predicts clinical outcome
Source: Nat Commun. 2020 Oct 5;11:4995. doi: 10.1038/s41467-020-18819-5 (PMC7536188; doi:10.1038/s41467-020-18819-5)
Supplement: Supplementary file 1 — Supplementary Information [file 41467_2020_18819_MOESM1_ESM.pdf]

Molecular stratification of endometrioid ovarian carcinoma predicts clinical outcome

Hollis *et al*

SUPPLEMENTARY INFORMATION

METHODS

SECTION 1: IMMUNOHISTOCHEMISTRY

1A. Immunohistochemistry for WT1

Immunohistochemistry (IHC) for Wilms' Tumour 1 (WT1) was performed on the Leica Bond III Autostainer using protocol F. WT1 IHC used 1:1000 dilution anti-human WT1 monoclonal mouse antibody clone 6F-H2 (DAKO). Nuclear WT1 expression in tumour cells was recorded as WT1 positive and those with complete absence of nuclear staining as WT1 negative. Positive nuclear staining of vascular endothelial cells served as internal controls.

1B. Immunohistochemistry for CK7 and CK20

Cytokeratin 7 (CK7) staining was performed using a 1:100 dilution of anti-human monoclonal mouse CK7 antibody clone RN7 (Leica). A WT1 positive high grade serous ovarian carcinoma tissue section was used as a positive control. Nuclear staining in tumour cells was considered CK7 positive.

Cytokeratin 20 (CK20) staining was performed using a 1:50 dilution of anti-human monoclonal mouse CK20 antibody clone KS20.8 (Leica). Normal stomach tissue was used as a positive control. Nuclear staining in tumour cells was considered CK20 positive.

1C. Immunohistochemistry for p53

IHC for tumour protein p53 (p53) was performed on the Leica BOND III Autostainer using protocol F. p53 IHC used a 1:50 dilution of the monoclonal mouse anti-human p53 antibody clone DO-7 (DAKO). p53 staining was recorded as aberrant (aberrant diffuse nuclear overexpression or aberrant null pattern) or wild-type (variable nuclear expression). Stromal cells served as an internal control.

1D. Immunohistochemistry for β-catenin

β-catenin IHC was performed using a human tissue microarray constructed from 0.8mm cores taken from EnOC tumour regions. IHC used a 1:100 dilution of the monoclonal mouse anti-human β-catenin antibody M353901-2 (Agilent) on the Leica BOND III Autostainer. Normal tonsil tissue was used as the control. β-catenin staining was recorded as aberrant (abnormal nuclear accumulation in tumour cells) or wild-type (membranous staining only). Stromal cells served as an internal control.

SECTION 2: WHOLE EXOME SEQUENCING

2A. Generation of sequence libraries and exome sequencing

Libraries were prepared from tumour DNA using the Illumina TruSeq Exome Library Prep kit (#FC-150-1002 - Illumina) according to the manufacturer's protocol using modifications for working with formalin-fixed paraffin-embedded (FFPE) material.

200ng of DNA was end-repaired to remove 3' and 5' overhangs and fragment length was optimised using sample purification beads. A single 'A' nucleotide was added to the 3' ends of the blunt fragments to prevent them from ligating to one another during subsequent adapter ligation, and a corresponding single 'T' nucleotide on the 3' end of the adapter provided a complementary overhang for ligating the adapter to the fragment. Multiple indexing adapters were then ligated to the ends of the cDNA to prepare them for hybridisation onto a flow cell, before 12 cycles of polymerase chain reaction (PCR) were used to selectively enrich adapter-bound DNA fragments and amplify DNA quantity. Libraries were quantified using the Qubit 2.0 Fluorometer and the Qubit DNA High Sensitivity (HS) assay (#Q32854 - ThermoFisher); size distribution of fragments was assessed using the Agilent Bioanalyser with the DNA HS Kit (#5067-4626 - Agilent).

DNA libraries containing unique indexes were combined into pools of 6 and target regions bound with capture probes. Streptavidin magnetic beads were then used to capture probes hybridised to the targeted regions of interest and a series of washes removed nonspecific binding from the beads. This process was repeated to ensure high specificity of the captured regions. Capture-enriched library was then purified before 8 cycles of PCR amplification and a final purification step to remove unwanted products.

Exome-captured sequencing library pools were quantified using the Qubit 2.0 Fluorometer and the Qubit DNA HS assay (#Q32854 - ThermoFisher) and the size distribution of fragments was assessed using the Agilent Bioanalyser with the DNA HS Kit (#5067-4626 - Agilent). Fragment size and quantity measurements were used to calculate molarity for each library pool.

Sequencing was performed using the NextSeq 500/550 High-Output v2 (150 cycle) Kit (# FC-404-2002) on the NextSeq 550 platform (Illumina Inc, #SY-415-1002).

2B. Mapping of sequenced reads

Base calling and quality were assessed using FASTQC. Data were processed with the bcbio-nextgen python toolkit for fully automated high throughput sequencing analysis (see https://github.com/bcbio/bcbio-nextgen for full documentation and informatic pipelines). Raw

sequence data was mapped to the hg38 genome build using the Burrows–Wheeler alignment algorithm 0.7.17 [1].

2C. Variant calling

Variant calling was carried out on mapped BAM files using a majority vote approach from three variant caller algorithms; VarDict [2], Mutect2 [3], Freebayes [4]. Filtering for FFPE and oxidation artifacts was applied using the GenomeAnalysisToolkit (GATK) (CollectSequencingArtifactMetrics and FilterByOrientationBias). Resulting variant call (VCF) files were analysed in R using the maftools package [5]. Datasets were filtered to remove common population variants using the 1000 Genomes reference datasets (1000 genomes phase 1 SNP and InDel dataset; http://www.internationalgenome.org/) and the Exome Aggregation consortium (ExAC) reference datasets (ExAC.0.3.GRCh38 : http://exac.broadinstitute.org/).

Variants of known function were flagged using the NCBI ClinVar database [6]; variants of unlikely functional significance were filtered using the Polymorphism Phenotyping (PolyPhen) [7] and Sorting Intolerant from Tolerant (SIFT) [8] prediction tools. Variants with <10% variant allele frequency or a read coverage of <20X were removed.

Microsatellite instability (MSI) scores were assigned based on the number of InDels detected in a given sample. Transitions and transversions were calculated using the titv function in maftools.

SECTION 3: ONCOGENIC PATHWAY ANALYSIS

Pathway analysis was performed using the OncogenicPathways function in the R package maftools. This highlighted PIK-AKT, WNT, RAS and NOTCH pathways as major altered networks (figure S3).

SECTION 4: TUMOUR COMPLEXITY SCORING

Variant allele frequency (VAF) densities across all genes were plotted for each sample to assess tumour genomic complexity; low complexity specimens, with a single driver event and associated outgrowth, were anticipated to display a single VAF peak. Conversely, highly complex tumours with multiple driver events, branched evolution and cell population expansion, would demonstrate multiple VAF peaks. Analysis was carried out using the inferHeterogeneity function in the R package maftools [5, 9]. Resulting mutant-allele tumour heterogeneity (MATH) scores represent the width of the VAF distribution.

SUPPLEMENTARY FIGURES

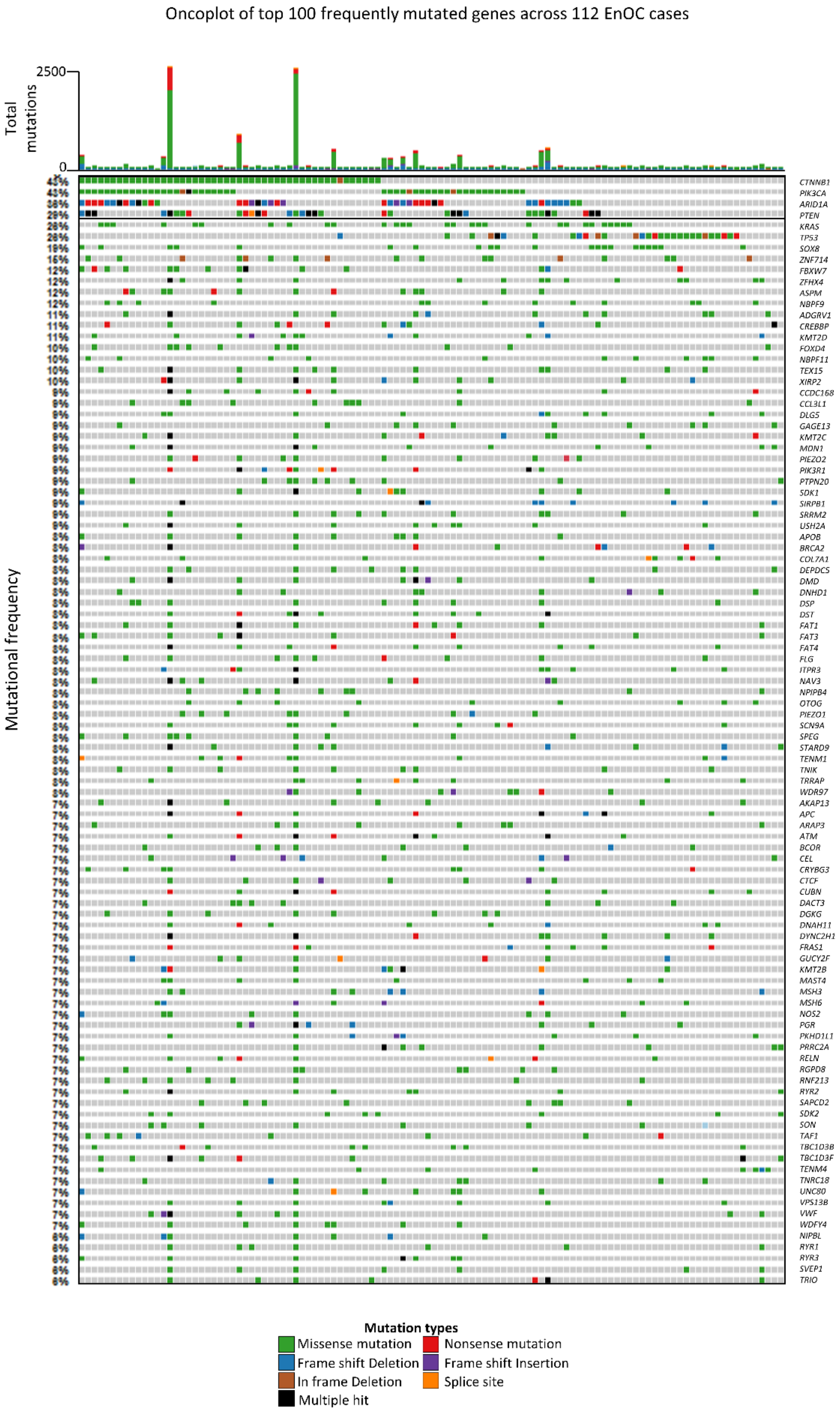

Figure S1. Oncoplot for the 100 most frequently mutated genes from whole exome analysis.

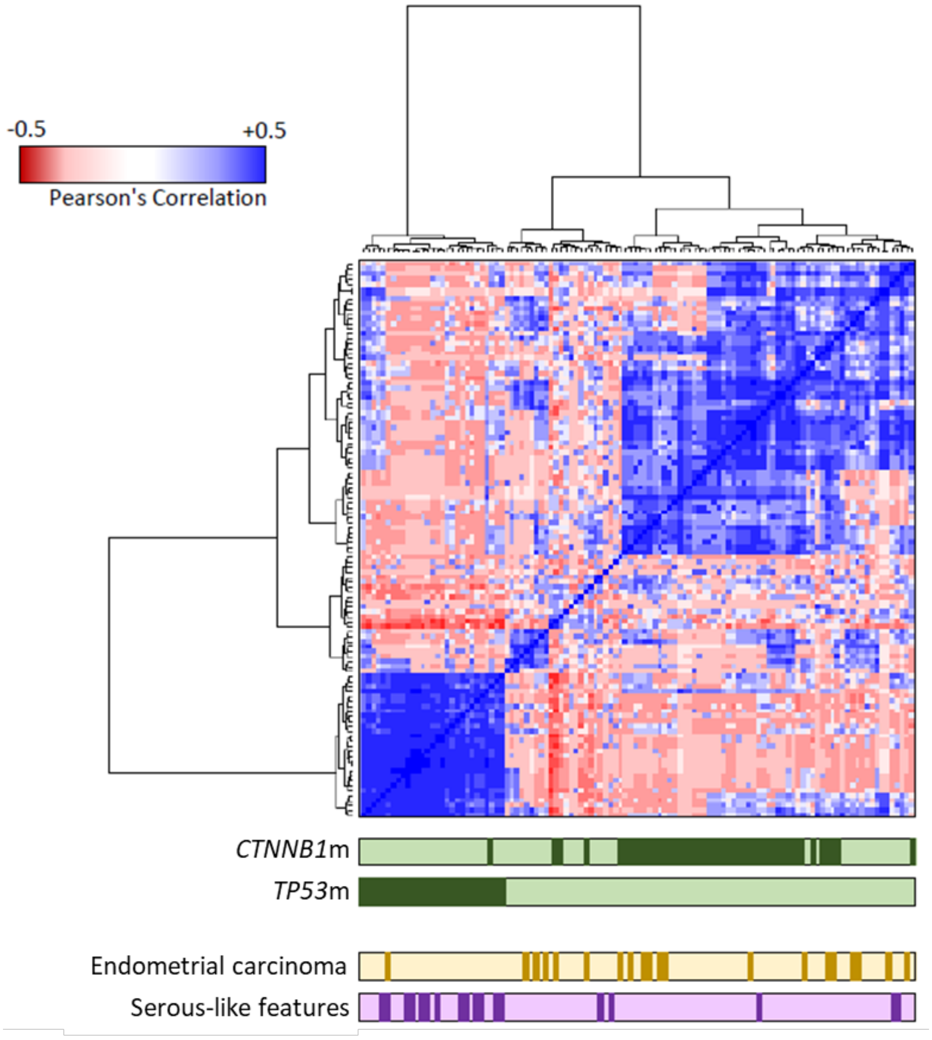

Figure S2. Unsupervised clustering of endometrioid ovarian carcinomas by patterns of mutation, annotated to highlight cases with concurrent endometrial cancer diagnosis or serous-like morphological features. Product-moment correlation scores between samples were calculated using binary matrices representing the status of most frequently mutated genes (1=mutant, 0=wild-type), yielding a matrix of quantified genomic correlation. These data were subject to hierarchical clustering using Euclidean distance and Ward’s linkage.

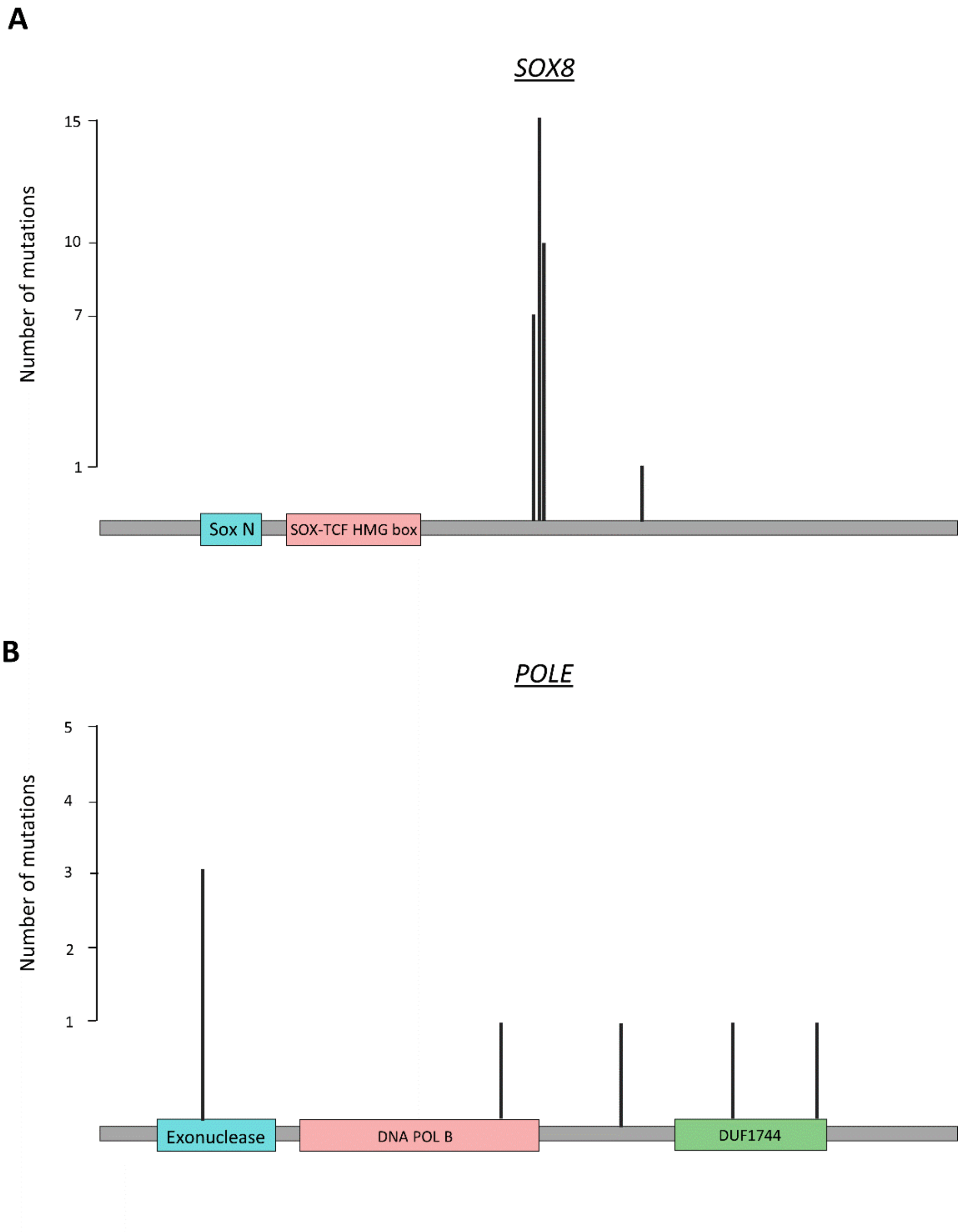

Figure S3. Mutation maps of *SOX8* and *POLE*. Lollipop plot of location of variants within (A) *SOX8* and (B) *POLE*, with known protein coding domains highlighted.

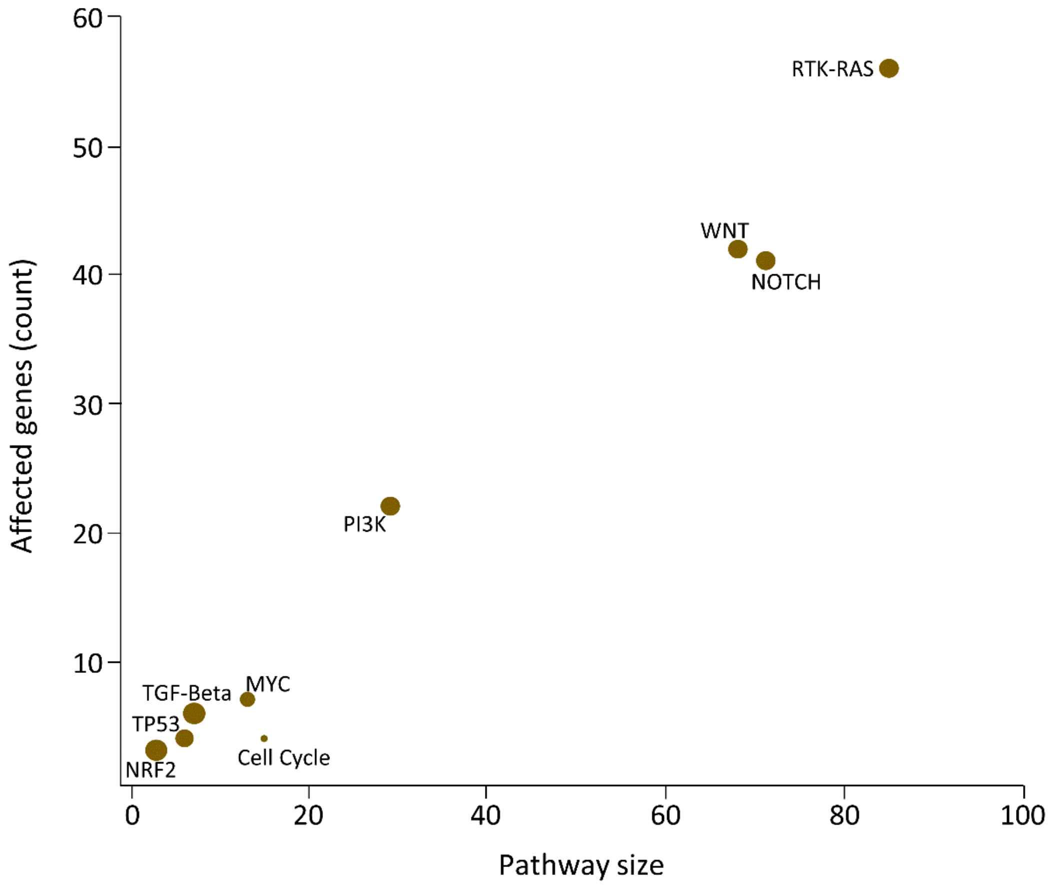

Figure S4. Scatter plot of the number of genes altered in oncogenic pathways versus total pathway size.

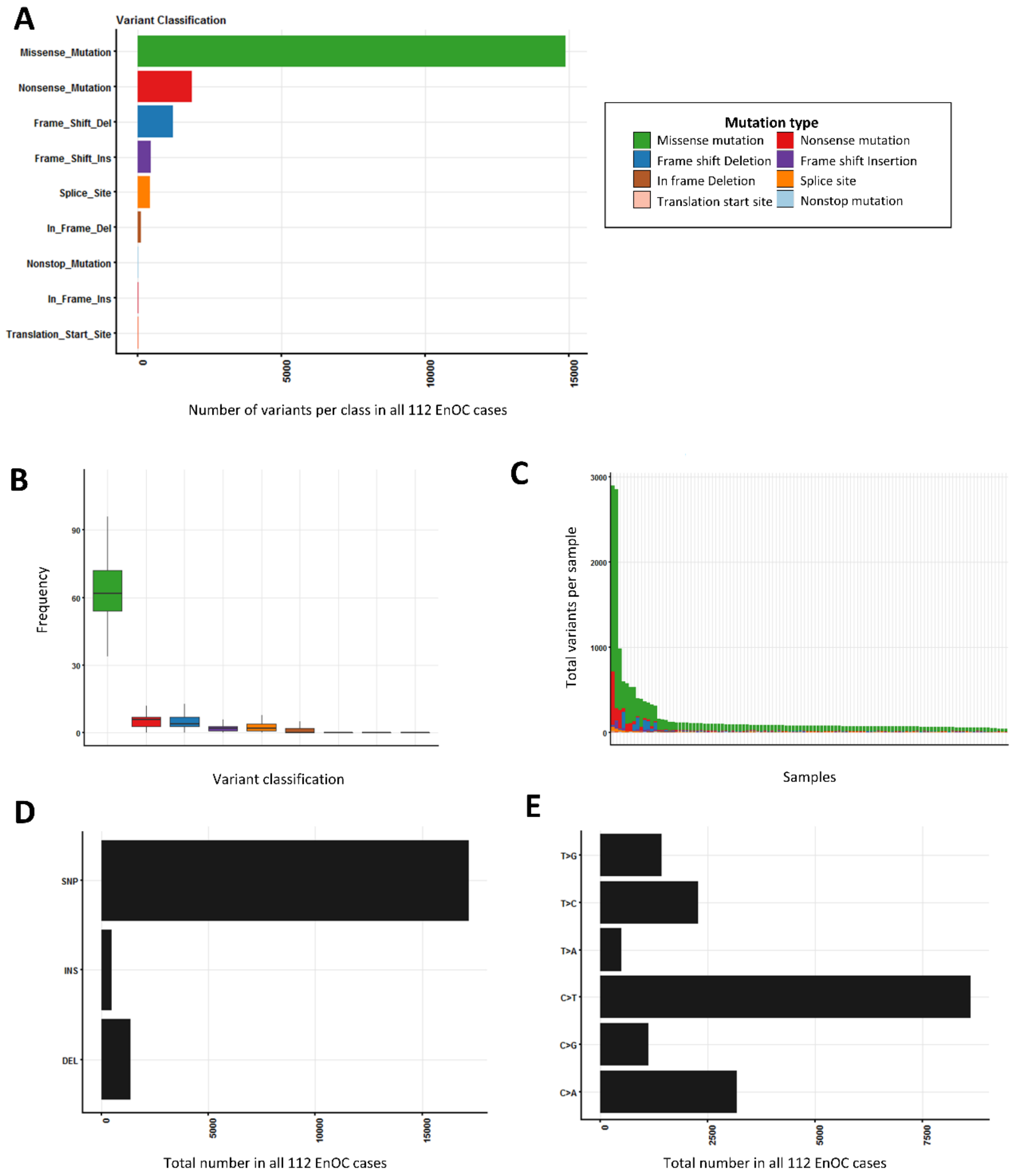

Figure S5. Whole exome variant call summary statistics for 112 EnOC cases. (A) Plot of the total number of variant types across 112 endometrioid ovarian carcinomas. (B) Box plot of the number of variants per sample for each classification. Boxes represent 1st to 3rd quartile, with the median labelled as the central line; whiskers extend to the data range from 1st and 3rd quartile +/-1.5 times the interquartile range. (C) Stacked plot of total variant count per sample. (D) Plot of variant types. (E) Summary of base changes across endometrioid ovarian carcinoma cases. SNP, single nucleotide polymorphism; INS, insertion; DEL, deletion.

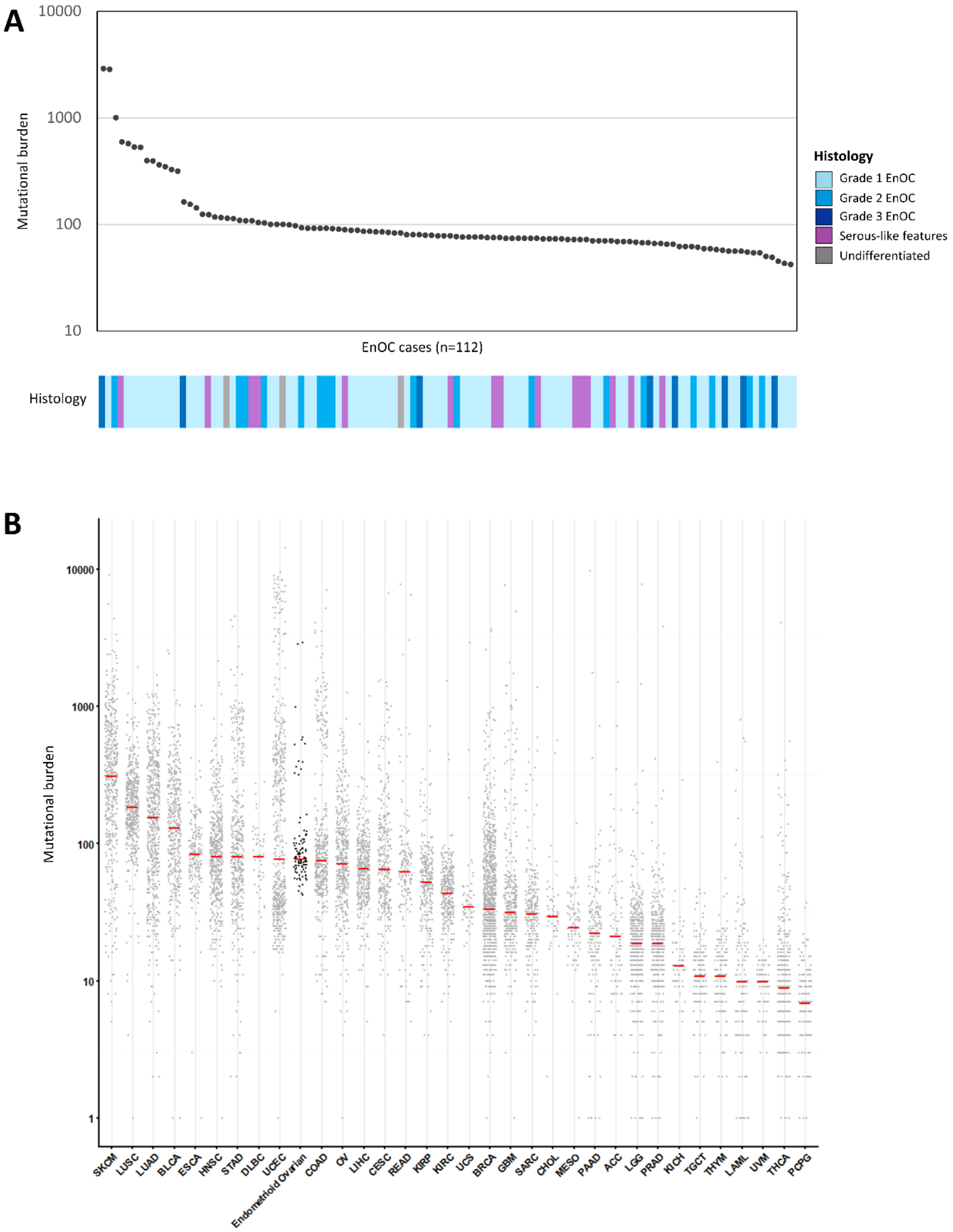

Figure S6. Tumour mutational burden. (A) Summary of tumour mutational burden across 112 endometrioid ovarian carcinoma samples. (B) Mutational burden in the endometroid ovarian carcinoma against 33 TCGA landmark cohort datasets. Individual dots represent each sample (grey = TCGA, black = our study). Red bar denotes median mutation count. SKCM: Skin cutaneous melanoma, LUSC: Lung squamous cell carcinoma, LUAD: Lung adenocarcinoma, BLCA: Bladder urothelial carcinoma, ESCA: Esophageal carcinoma, HNSC: Head and neck squamous cell carcinoma, STAD: Stomach adenocarcinoma, DLBC: Lymphoid neoplasm diffuse large B-cell lymphoma, UCEC: Uterine corpus endometrial carcinoma, COAD: Colon adenocarcinoma, OV: Ovarian serous

cystadenocarcinoma, LIHC: Liver hepatocellular carcinoma, CESC: Cervical squamous cell carcinoma and endocervical adenocarcinoma, READ: Rectum adenocarcinoma, KIRP: Kidney renal papillary cell carcinoma, KIRC: Kidney renal clear cell carcinoma, UCS: Uterine carcinosarcoma, BRCA: Breast invasive carcinoma, GBM: Glioblastoma multiforme, SARC: Sarcoma, CHOL: Cholangiocarcinoma, MESO: Mesothelioma, PAAD: Pancreatic adenocarcinoma, ACC: Adrenocortical carcinoma, LGG: Brain lower grade glioma, PRAD: Prostate adenocarcinoma, KICH: Kidney chromophobe, TGCT: Testicular germ cell tumors, THYM: Thymoma, AML: Acute myeloid leukemia, UVM: Uveal melanoma, THCA: Thyroid carcinoma, PCPG: Pheochromocytoma and paraganglioma. EnOC, Endometrioid ovarian carcinoma.

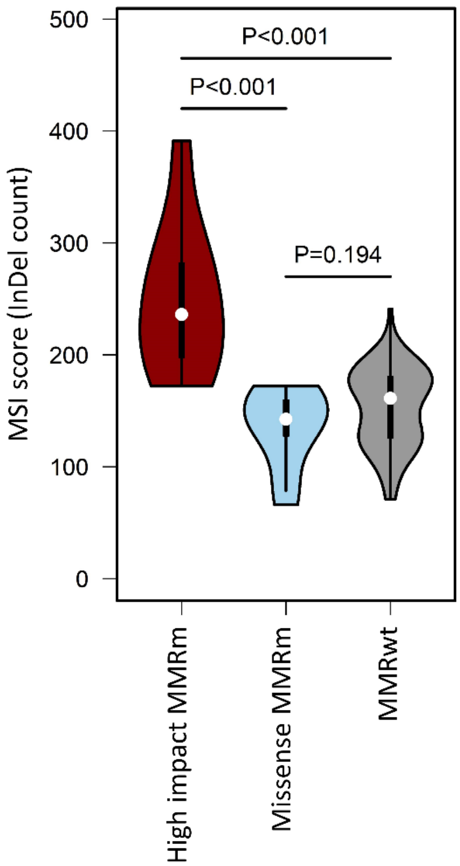

Figure S7. Violin plot of MSI score (number of InDels in a given tumour) split by mismatch repair mutation status. High impact MMRm, n=10; missense MMRm, n=10; MMRwt, n=92 .Comparisons were made using the two-sided Mann-Whitney U test without adjustment for multiplicity of testing (high impact MMRm vs missense MMRm P=0.0002; missense MMRm vs MMRwt, P=0.1936; high impact MMRm vs MMRwt, P<0.0001). MMR, mismatch repair; high impact MMRm: frameshifting InDels, nonsense and splice site mutations; MMRm, MMR mutation; MMRwt, MMR wild-type.

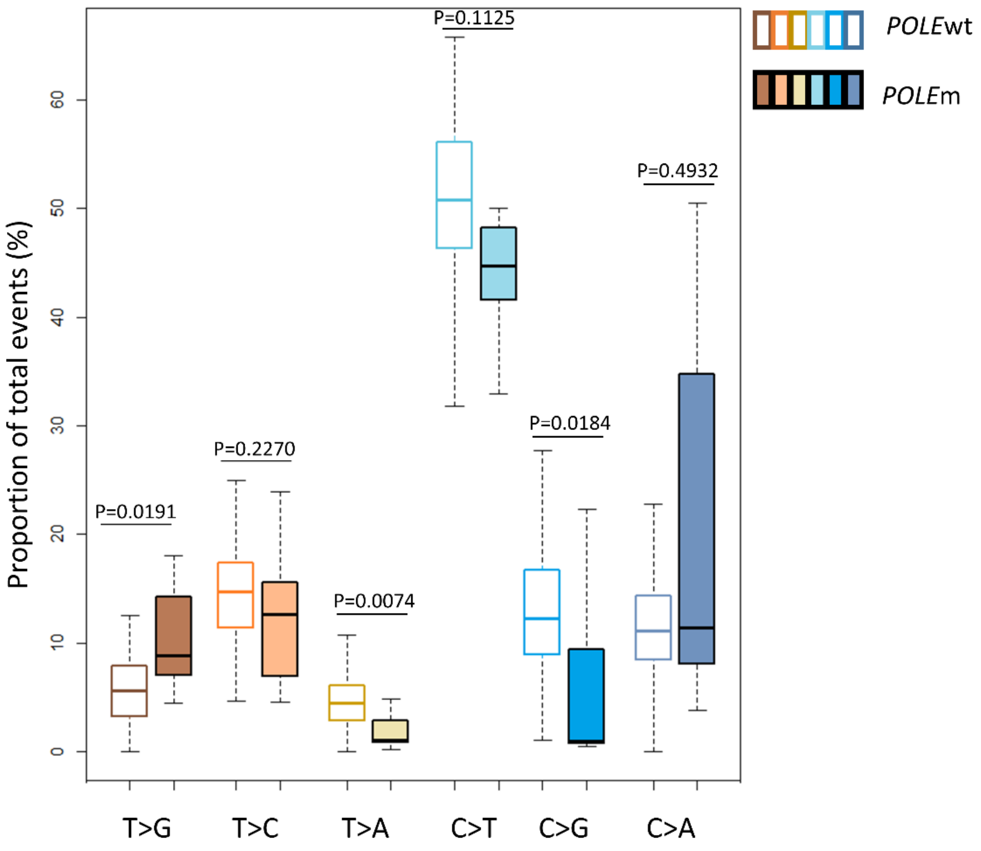

Figure S8. Boxplots of base substitutions between *POLE*wt (n=105) and *POLE*m (n=7) tumours. Boxes represent 1st to 3rd quartile, with the median labelled as the central line; whiskers extend to the data range from 1st and 3rd quartile +/-1.5 times the interquartile range. Comparisons were made using two-sided Mann Whitney-U tests. Labelled P values are not adjusted for multiple testing. M, mutant; wt, wild-type; ns, not significant.

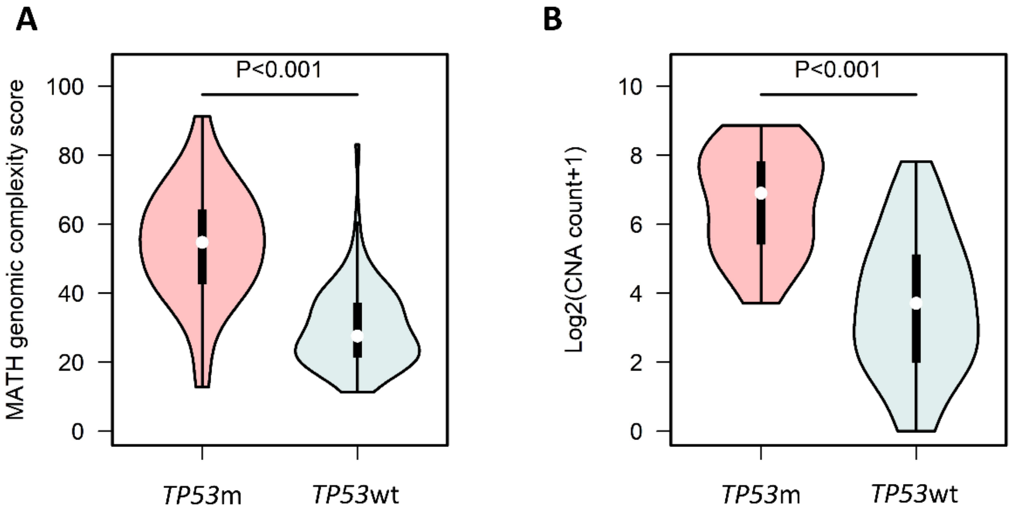

Figure S9. Genomic complexity and copy number alteration burden in *TP53* mutant (*TP53*m) cases. Violin plots displaying (A) genomic complexity and (B) copy number alterations in cases by *TP53* mutation status. *TP53*m, n=29; *TP53* wild-type (*TP53*wt), n=83. Comparisons were made using the two-sided Mann-Whitney U test without adjusting for multiplicity of testing (*TP53*m vs *TP53*wt MATH score, P<0.001; *TP53*m vs *TP53*wt copy number alteration count, P<0.001). MATH, mutant-allele tumour heterogeneity; CNA, copy number alteration.

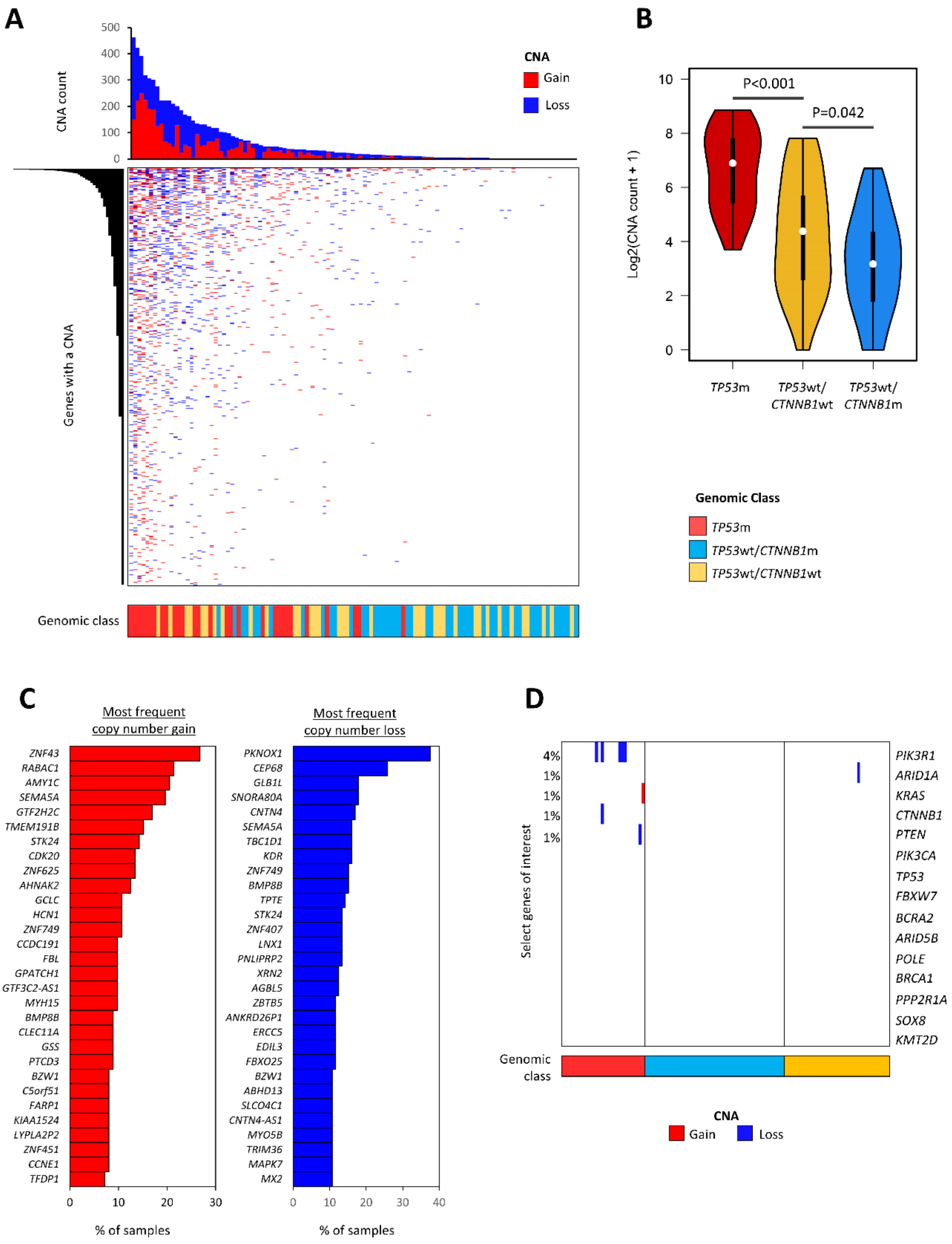

Figure S10. Analysis of copy number alterations in endometrioid ovarian carcinoma. (A) Copy number landscape across cases (red=gain, blue=loss). (B) Number of copy number alteration events in genomic subtypes defined by *TP53* and *CTNNB1* status. Comparisons were made using the two-sided Mann-Whitney U test without adjusting for multiplicity of testing (*TP53*m vs *TP53*wt/*CTNNB1*wt, P<0.001;

*TP53*wt/*CTNNB1*wt vs *TP53*wt/*CTNNB1*m, P=0.042). (C) Top 30 most frequent gene targets of copy number alteration (red=gain, blue=loss). (D) Copy number alterations over frequently mutated genes SNV, single nucleotide variant; CNA, copy number alteration.

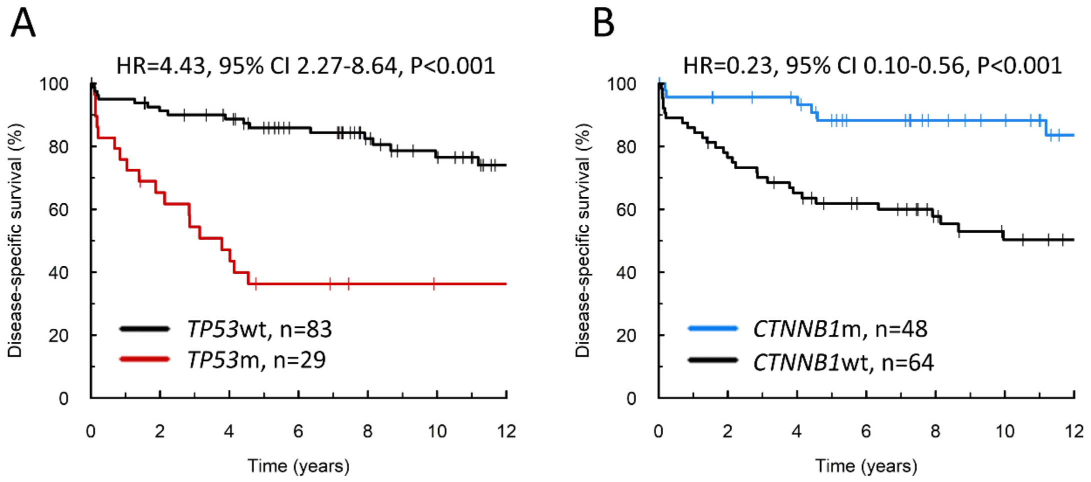

Figure S11. Disease-specific survival of endometrioid ovarian carcinoma cases. (A) Impact of *TP53* status and (B) impact of *CTNNB1* status. Comparisons were made using Cox proportional hazards regression models (*TP53*m vs *TP53*wt, P<0.0001; *CTNNB1*m vs *CTNNB1*wt, P<0.0001). Labelled P values are not adjusted for multiple testing. HR, hazard ratio; m, mutant; wt, wild-type.

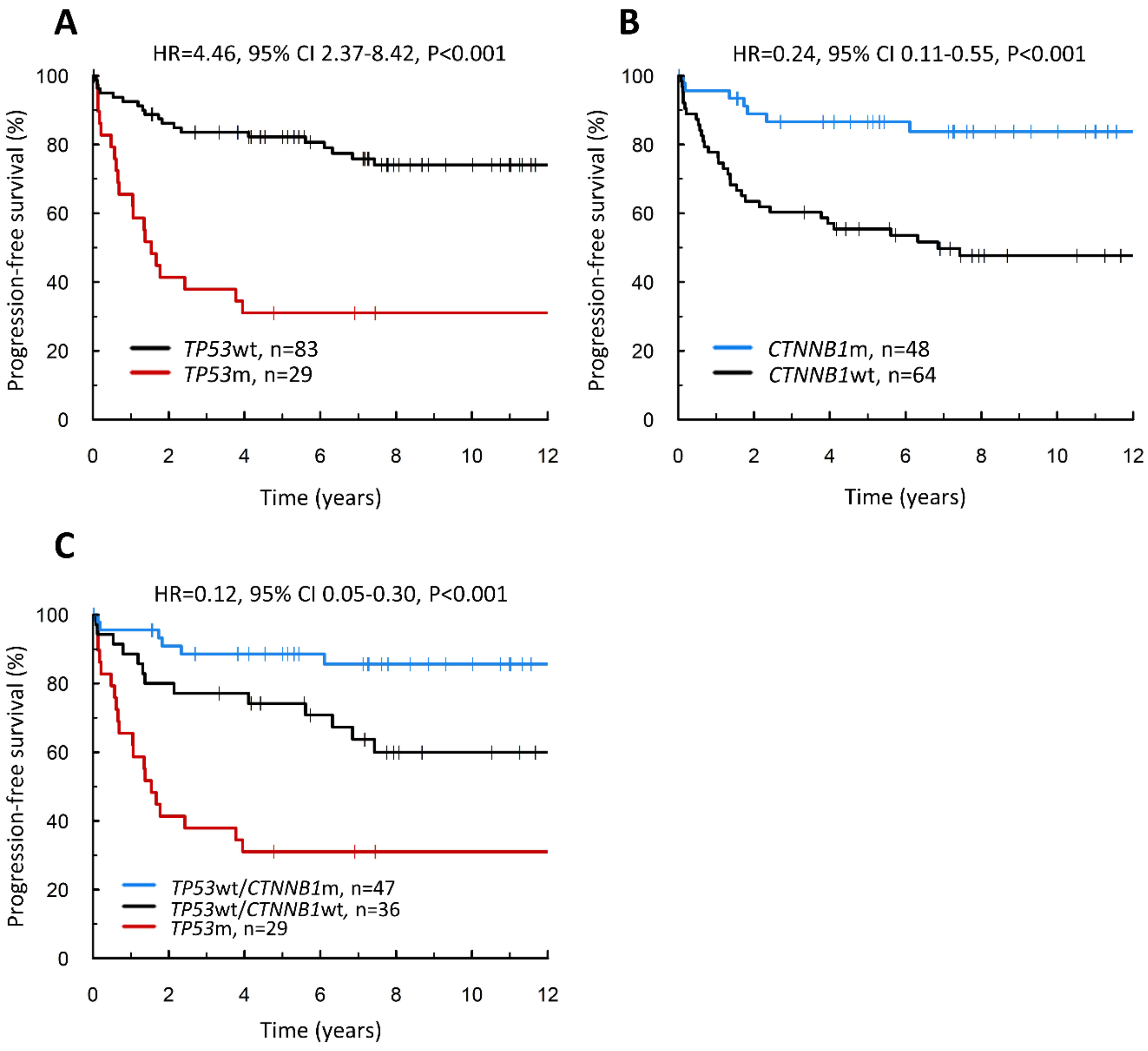

Figure S12. Progression-free survival of patient subgroups. (A) Impact of *TP53*m, (B) impact of *CTNNB1*m and (C) combined *TP53*m and *CTNNB1*m; in (C) the labelled hazard ratio (HR) refers to *TP53*wt/*CTNNB1*m versus *TP53*m (blue versus red lines; P<0.001). In addition, HR for *TP53*wt/*CTNNB1*m versus *TP53*wt/*CTNNB1*wt (blue versus black lines) = 0.33, 95% CI 0.13-0.87; and for *TP53*wt/*CTNNB1*wt versus *TP53*m (black versus red lines) = 0.37, 95% CI 0.18-0.74. Comparisons were made using Cox proportional hazards regression models. Labelled P values are not adjusted for multiple testing. m, mutant; wt, wild-type.

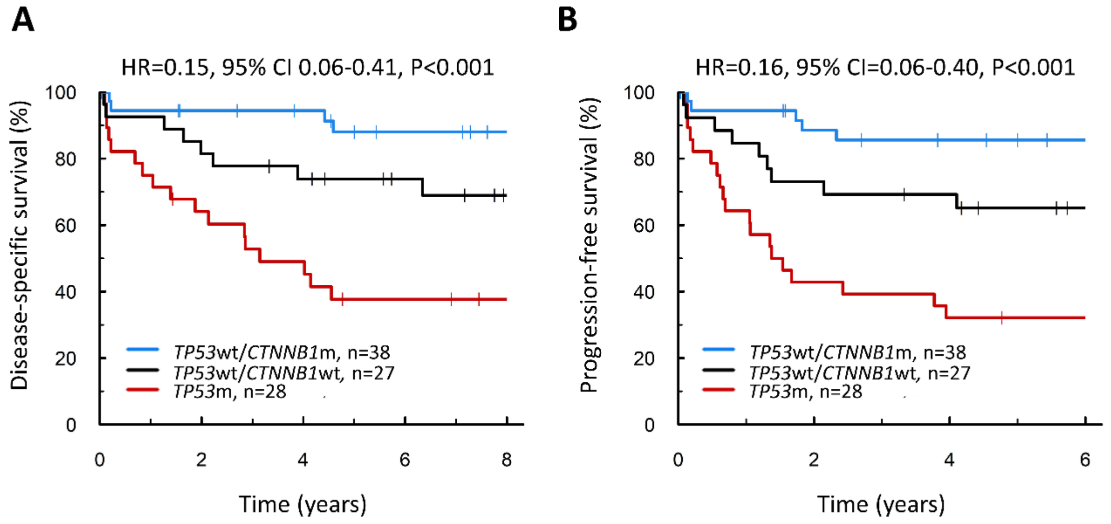

Figure S13. Survival analysis of endometrioid ovarian carcinoma after exclusion of cases with concurrent endometrial carcinoma. (A) Disease-specific survival; the labelled hazard ratio (HR) refers to *TP53*wt/*CTNNB1*m versus *TP53*m (blue versus red lines; P<0.001). In addition, HR for *TP53*wt/*CTNNB1*m versus *TP53*wt/*CTNNB1*wt (blue versus black lines) = 0.29, 95% CI 0.10-0.84, and *TP53*wt/*CTNNB1*wt versus *TP53*m (black versus red lines) = 0.52, 95% CI 0.24-1.11. (B) Progression-free survival; the labelled HR refers to *TP53*wt/*CTNNB1*m versus *TP53*m (blue versus red lines; P<0.001). In addition, HR for *TP53*wt/*CTNNB1*m versus *TP53*wt/*CTNNB1*wt (blue versus black lines) = 0.34, 95% CI 0.13-0.92, and *TP53*wt/*CTNNB1*wt versus *TP53*m (black versus red lines) = 0.47, 95% CI 0.22-0.99. Comparisons were made using Cox proportional hazards regression models. Labelled P values are not adjusted for multiple testing. m, mutant; wt, wild-type.

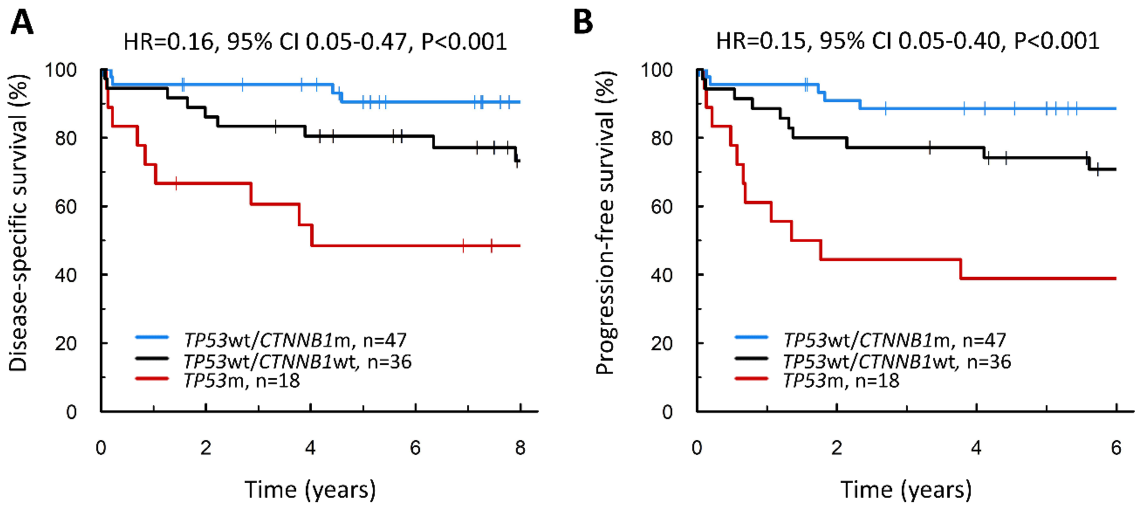

Figure S14. Survival analysis of endometrioid ovarian carcinoma after exclusion of *TP53*m cases without mutation in *PTEN*/*ARID1A*/*KRAS*/*CTNNB1*/*PIK3CA*/*SOX8*. (A) Disease-specific survival; the labelled hazard ratio (HR) refers to *TP53*wt/*CTNNB1*m versus *TP53*m (blue versus red lines; P<0.001). In addition, HR for *TP53*wt/*CTNNB1*m versus *TP53*wt/*CTNNB1*wt (blue versus black lines) = 0.31, 95% CI 0.11-0.88, and *TP53*wt/*CTNNB1*wt versus *TP53*m (black versus red lines) = 0.51, 95% CI 0.22-1.22. (B) Progression-free survival; the labelled HR refers to *TP53*wt/*CTNNB1*m versus *TP53*m (blue versus red lines; P<0.001). In addition, HR for *TP53*wt/*CTNNB1*m versus *TP53*wt/*CTNNB1*wt (blue versus black lines) = 0.33, 95% CI 0.13-0.87, and *TP53*wt/*CTNNB1*wt versus *TP53*m (black versus red lines) = 0.45, 95% CI 0.20-1.00. Labelled P values are not adjusted for multiple testing. m, mutant; wt, wild-type.

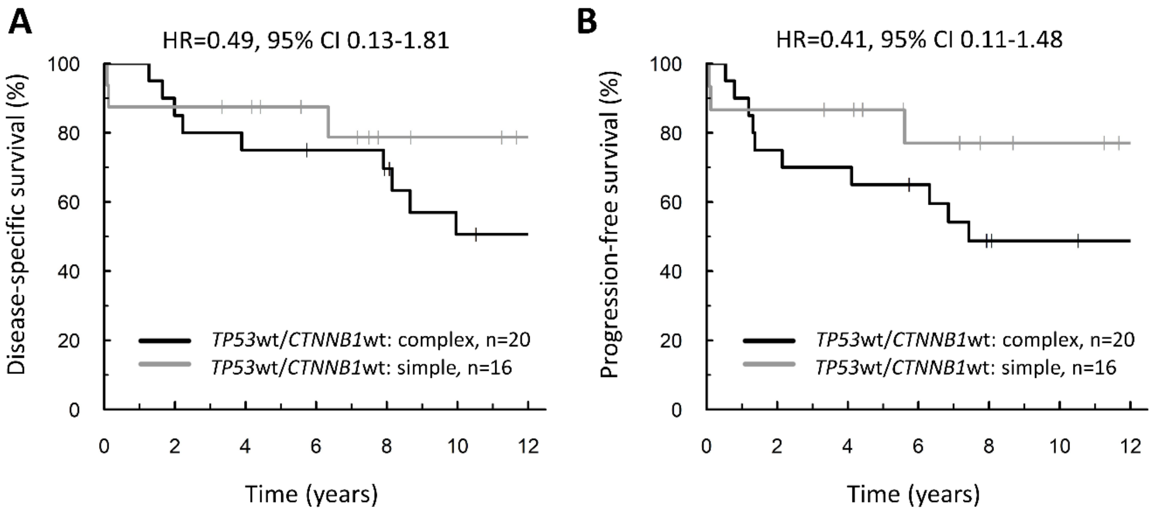

Figure S15. Impact of MATH genomic complexity score in the *TP53*wt/*CTNNB1*wt subgroup. (A) Disease-specific survival, (B) Progression-free survival. Simple versus complex split at median MATH score for EnOCs with a single variant allele frequency peak (figure 4Aiii). HR, hazard ratio; wt, wild-type

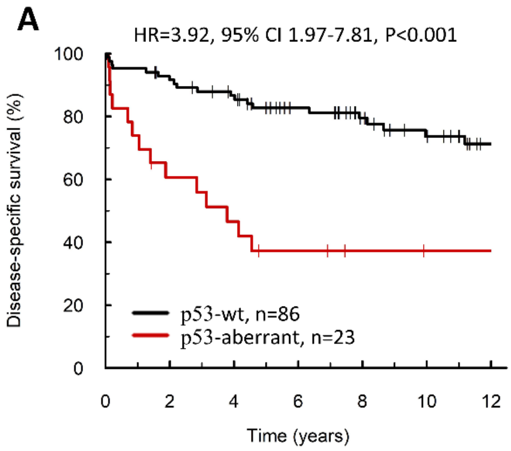

	p53-aberrant	p53-wt
*TP53*m	22	5
*TP53*wt	1	81
Chi-square test P<0.0001		
IHC sensitivity: 81.5%; IHC specificity: 95.7%		

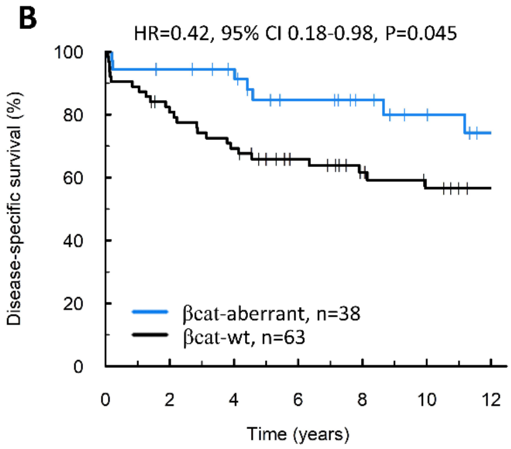

	βcat-aberrant	βcat-wt
*CTNNB1*m	32	12
*CTNNB1*wt	6	51
Chi-square test P<0.0001		
IHC sensitivity: 72.7%; IHC specificity: 84.2%		

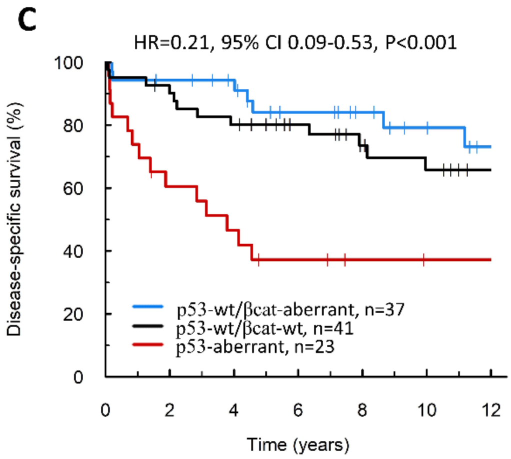

	p53-aberrant	p53-wt/ βcat-wt	p53-wt/ βcat-aberrant
*TP53*m	22	4	1
*TP53*wt/ *CTNNB1*wt	1	25	5
*TP53*wt/ *CTNNB1*m	0	12	31
Chi-square test P<0.0001			

Figure S15. Disease-specific survival of groups defined by immunohistochemistry. (A) Impact of tumour protein p53 and (B) β-catenin (β-cat). (C) Recapitulation of the PRISTINE algorithm subgroups by immunohistochemistry; the labelled HR represents comparison of the p53-wt/β-cat-aberrant group with the p53-aberrant group (blue versus red lines); in addition, HR for p53-wt/βcat-wt versus p53-aberrant = 0.32, 95% CI 0.15-0.70 (black versus red lines); and HR for p53-wt/βcat-aberrant versus p53-wt/βcat-wt = 0.67, 95% CI 0.26-1.69 (blue versus black lines). Comparisons were made using Cox proportional hazards regression models and Chi-square tests. Labelled P values are not adjusted for

multiple testing. HR, hazard ratio; aberrant, aberrant protein expression pattern; wt, wild-type protein expression pattern.

SUPPLEMENTARY TABLES

Table S1. *BRCA1* and *BRCA2* mutations detected in endometrioid ovarian carcinoma cases

Gene	Variant Classification	Variant	ID	Protein change	ClinVar Pathogenicity	Previously reported in germline	ClinVar Annotation	Note
BRCA1	Frame Shift Del	c.2681 2682del	rs80357971	p.Lys894ThrfsTer8	pathogenic	yes	https://www.ncbi.nlm.nih.gov/clinvar/variation/17667/	
BRCA1	Missense	c.1897C>T	rs80356902	p.Pro633Ser	conflicting interpretation of pathogenicity	yes	https://www.ncbi.nlm.nih.gov/clinvar/variation/54387/	
BRCA1	Frame Shift Del	c.1961del	rs80357522	p.Lys654SerfsTer47	pathogenic	yes	https://www.ncbi.nlm.nih.gov/clinvar/variation/37438/	
BRCA1	Frame Shift Del	c.3756 3759del	rs80357868	p.Ser1253ArgfsTer10	pathogenic	yes	https://www.ncbi.nlm.nih.gov/clinvar/variation/17673/	Known germline mutation: reported on routine clinical sequencing
BRCA1	Splice Site	c.80+1G>T	rs80358010		pathogenic	yes	https://www.ncbi.nlm.nih.gov/clinvar/variation/125517/	
BRCA2	Frame Shift Del	c.4876 4877del	novel	p.Asn1626SerfsTer12	NA		NA	
BRCA2	Nonsense	c.37G>T	rs80358622	p.Glu13Ter	pathogenic	yes	https://www.ncbi.nlm.nih.gov/clinvar/variation/51527/	
BRCA2	Missense	c.1455G>T	novel	p.Lys485Asn	NA	no - novel	NA	
BRCA2	Frame Shift Ins	c.6129dup	novel	p.Gly2044ArgfsTer5	NA	no - novel	NA	Other frameshifting variant(s) at this position previously reported as pathogenic
BRCA2	Missense	c.2585A>T	novel	p.Lys862Ile	NA	no - novel	NA	
BRCA2	Nonsense	c.2659G>T	novel	p.Glu887Ter	NA	no - novel	NA	
BRCA2	Nonsense	c.5782G>T	rs56253082	p.Glu1928Ter	pathogenic	yes	https://www.ncbi.nlm.nih.gov/clinvar/variation/37996/	
BRCA2	Missense	c.9428T>G	novel	p.Phe3143Cys	NA	no - novel	NA	
BRCA2	Missense	c.4436G>T	novel	p.Ser1479Ile	NA	no - novel	NA	
BRCA2	Missense	c.8587G>C	novel	p.Glu2863Gln	NA	no - novel	NA	
BRCA2	Nonsense	c.2409T>G	rs80358504	p.Tyr803Ter	pathogenic	yes	https://www.ncbi.nlm.nih.gov/clinvar/variation/37784/	Known germline mutation: reported on routine clinical sequencing
BRCA2	Frame Shift Del	c.4876 4877del	novel	p.Asn1626SerfsTer12	NA	no - novel	NA	Other frameshifting variant(s) at this position previously reported as pathogenic
BRCA2	Nonsense	c.6952C>T	rs80358920	p.Arg2318Ter	pathogenic	yes	https://www.ncbi.nlm.nih.gov/clinvar/variation/38076/	

NA, not applicable

Table S2. *TP53* mutation status versus discrete variant allele frequency peak counts. The labelled P-value is not adjusted for multiple testing.

	***TP53*m**		***TP53*wt**	
**VAF peaks**	n	%	n	%
**1**	4	13.8	45	54.2
**2**	17	58.6	34	41.0
**≥3**	8	27.6	4	4.8
**total**	29		83	
**Chisq P<0.001; 1 peak versus >1 peak**				

m, mutant; wt, wild-type; VAF, variant allele frequency

Table S3. Univariable analysis of survival, with adjustment for multiplicity of testing. DSS, disease-specific survival; PFS, progression-free survival

			HR	95% CI	P-value	P adj.
TP53	DSS	mutant	4.43	2.27-8.64	<0.001	<0.001
		wild-type	-	-	-	-
	PFS	mutant	4.46	2.37-8.42	<0.001	<0.001
		wild-type	-	-	-	-
CTNNB1	DSS	mutant	0.23	0.10-0.56	0.001	0.010
		wild-type	-	-	-	-
	PFS	mutant	0.24	0.11-0.55	<0.001	0.005
		wild-type	-	-	-	-
PIK3CA	DSS	mutant	0.76	0.38-1.51	0.439	1.00
		wild-type	-	-	-	-
	PFS	mutant	0.67	0.34-1.30	0.230	1.00
		wild-type	-	-	-	-
ARID1A	DSS	mutant	0.48	0.22-1.06	0.069	0.552
		wild-type	-	-	-	-
	PFS	mutant	0.72	0.36-1.42	0.341	1.00
		wild-type	-	-	-	-
PTEN	DSS	mutant	0.48	0.20-1.15	0.098	0.7832
		wild-type	-	-	-	-
	PFS	mutant	0.59	0.27-1.28	0.178	1.00
		wild-type	-	-	-	-
KRAS	DSS	mutant	0.48	0.20-1.15	0.099	0.794
		wild-type	-	-	-	-
	PFS	mutant	0.44	0.18-1.04	0.062	0.495
		wild-type	-	-	-	-
MMR	DSS	mutant	0.43	0.13-1.42	0.167	1.00
		wild-type	-	-	-	-
	PFS	mutant	0.65	0.25-1.65	0.362	1.00
		wild-type	-	-	-	-
POLE	DSS	mutant	0.38	0.05-2.76	0.337	1.00

		wild-type	-	-	-	-
	PFS	mutant	0.73	0.18-3.03	0.663	1.00
		wild-type	-	-	-	-

DSS, disease-specific survival. PFS, progression-free survival; HR, hazard ratio; CI confidence interval; P adj., Bonferroni-adjusted P-value

Table S4. Clinicopathological features of endometrioid ovarian carcinoma genomic subtypes.

	*TP53*m		*TP53*wt/*CTNNB1*m		*TP53*wt/CTNNB1wt	
	n	%	n	%	n	%
**Cases**	29		47		36	
**Concurrent endometrial cancer**	1	3.4	9	19.1	9	25.0
**Age median (years)**	61	32-79	57	28-88	57	37-75
**FIGO stage**						
**I**	8	27.6	24	53.3	15	41.7
**II**	7	24.1	17	37.8	15	41.7
**III**	8	27.6	3	6.7	4	11.1
**IV**	6	20.7	1	2.2	2	5.6
**NA**	0		2		0	
**RD following debulking**						
**Zero macroscopic RD**	15	55.6	41	91.1	26	78.8
**Macroscopic RD**	12	44.4	4	8.9	7	21.2
**NA**	2		2		3	

RD, residual disease; m, mutant; wt, wild-type; NA, not available

Table S5. Multivariable disease-specific survival analysis by *TP53* mutation status. Labelled P values represent the output from multivariable analysis without further adjustment for multiple testing.

DSS		mHR	95% CI	P-value
TP53	*TP53*m	2.62	1.09-6.25	0.031
	*TP53*wt	-	-	-
**FIGO stage at diagnosis**	I/II	0.2	0.08-0.50	<0.001
	III/IV	-	-	-
**RD following debulking**	Zero macroscopic RD	0.21	0.08-0.54	0.001
	Macroscopic RD	-	-	-
**Diagnosis period**	1980s	-	-	-
	1990s	0.66	0.25-1.74	0.401
	2000s	0.37	0.13-1.10	0.074
	2010s	0.49	0.10-2.55	0.399
**Age**	years	1.02	0.98-1.05	0.369

DSS, disease-specific survival; mHR, multivariable hazard ratio; m, mutant; wt, wild-type; RD, residual disease

Table S6. Multivariable progression-free survival analysis by *TP53* mutation status. Labelled P values represent the output from multivariable analysis without further adjustment for multiple testing.

**PFS**		**mHR**	**95% CI**	**P-value**
TP53	*TP53*m	2.84	1.27-6.31	0.011
	*TP53*wt	-	-	-
**FIGO stage at diagnosis**	I/II	0.18	0.07-0.47	<0.001
	III/IV	-	-	-
**RD following debulking**	Zero macroscopic RD	0.30	0.12-0.75	0.010
	Macroscopic RD	-	-	-
**Diagnosis period**	1980s	-	-	-
	1990s	0.57	0.22-1.48	0.248
	2000s	0.32	0.11-0.88	0.028
	2010s	0.55	0.15-1.99	0.360
**Age**	years	1.02	0.98-1.05	0.327

PFS, progression-free survival; mHR, multivariable hazard ratio; m, mutant; wt, wild-type; RD, residual disease

Table S7. Multivariable disease-specific survival analysis of *CTNNB1* mutation status. Labelled P values represent the output from multivariable analysis without further adjustment for multiple testing.

DSS		mHR	95% CI	P-value
CTNNB1	*CTNNB1*m	0.31	0.12-0.81	0.017
	*CTNNB1*wt	-	-	-
**FIGO stage at diagnosis**	I/II	0.12	0.05-0.33	<0.001
	III/IV	-	-	-
**RD following debulking**	Zero macroscopic RD	0.32	0.12-0.86	0.023
	Macroscopic RD	-	-	-
**Diagnosis period**	1980s	-	-	-
	1990s	0.65	0.26-1.66	0.370
	2000s	0.29	0.10-0.89	0.030
	2010s	0.48	0.10-2.47	0.383
**Age**	years	1.02	0.99-1.06	0.154

DSS, disease-specific survival; mHR, multivariable hazard ratio; m, mutant; wt, wild-type; RD, residual disease

Table S8. Multivariable progression-free survival analysis of *CTNNB1* mutation status. Labelled P values represent the output from multivariable analysis without further adjustment for multiple testing.

PFS		mHR	95% CI	P-value
CTNNB1	*CTNNB1*m	0.29	0.12-0.69	0.006
	*CTNNB1*wt	-	-	-
**FIGO stage at diagnosis**	I/II	0.11	0.04-0.29	<0.001
	III/IV	-	-	-
**RD following debulking**	Zero macroscopic RD	0.43	0.17-1.11	0.080
	Macroscopic RD	-	-	-
**Diagnosis period**	1980s	-	-	-
	1990s	0.58	0.23-1.43	0.234
	2000s	0.25	0.09-0.72	0.010
	2010s	0.57	0.16-2.04	0.391
**Age**	years	1.02	0.99-1.06	0.153

PFS, progression-free survival; mHR, multivariable hazard ratio; m, mutant; wt, wild-type; RD, residual disease

Table S9. Impact of genomic complexity on survival outcome. Labelled P values are not adjusted for multiple testing.

			HR	95% CI	P-value
**VAF peaks**	DSS	1 peak	-	-	-
		≥2 peaks	2.39	1.12-5.10	0.025
	PFS	1 peak	-	-	-
		≥2 peaks	2.22	1.11-4.47	0.025
**MATH score (continuous)**	DSS	score	1.03	1.02-1.05	<0.001
	PFS	score	1.03	1.02-1.05	<0.001
**MATH score (high versus low)**	DSS	>median	3.88	1.76-8.55	<0.001
		≤median	-	-	-
	PFS	>median	4.00	1.89-8.44	<0.001
		≤median	-	-	-

HR, hazard ratio; 95% CI, 95% confidence interval; DSS, disease-specific survival; PFS, progression-free survival; VAF, variant allele frequency; MATH, mutant allele tumour heterogeneity

Supplementary References

1. Li, H. & Durbin, R. Fast and accurate long-read alignment with Burrows-Wheeler transform. *Bioinformatics* **26**, 589-595 (2010).
2. Lai, Z. et al. VarDict: a novel and versatile variant caller for next-generation sequencing in cancer research. *Nucleic Acids Res* **44**, e108 (2016).
3. Cibulskis, K. et al. Sensitive detection of somatic point mutations in impure and heterogeneous cancer samples. *Nat Biotechnol* **31**, 213-219 (2013).
4. Garrison E, Marth G. Haplotype-based variant detection from short-read sequencing. Preprint at https://arxiv.org/abs/1207.3907 (2012).
5. Mayakonda, A., Lin, D.C., Assenov, Y., Plass, C. & Koeffler, H.P. Maftools: efficient and comprehensive analysis of somatic variants in cancer. *Genome Res* **28**, 1747-1756 (2018).
6. Landrum, M.J. et al. ClinVar: public archive of relationships among sequence variation and human phenotype. *Nucleic Acids Res* **42**, D980-985 (2014).
7. Adzhubei, I.A. et al. A method and server for predicting damaging missense mutations. *Nat Methods* **7**, 248-249 (2010).
8. Ng, P.C. & Henikoff, S. SIFT: Predicting amino acid changes that affect protein function. *Nucleic Acids Res* **31**, 3812-3814 (2003).
9. Mroz, E.A. & Rocco, J.W. MATH, a novel measure of intratumor genetic heterogeneity, is high in poor-outcome classes of head and neck squamous cell carcinoma. *Oral Oncol* **49**, 211-215 (2013).
