# Supplementary material for: Molecular stratification of endometrioid ovarian carcinoma predicts clinical outcome
Source: Nat Commun. 2020 Oct 5;11:4995. doi: 10.1038/s41467-020-18819-5 (PMC7536188; doi:10.1038/s41467-020-18819-5)
Supplement: Supplementary file 2 — Reporting Summary [file 41467_2020_18819_MOESM2_ESM.pdf]

nature research

Corresponding author(s): C Simon Herrington

Last updated by author(s): Sep 11, 2020

Reporting Summary

Nature Research wishes to improve the reproducibility of the work that we publish. This form provides structure for consistency and transparency in reporting. For further information on Nature Research policies, see Authors & Referees and the Editorial Policy Checklist .

Statistics

For all statistical analyses, confirm that the following items are present in the figure legend, table legend, main text, or Methods section.

n/a	Confirmed	
☐	☒	The exact sample size (*n*) for each experimental group/condition, given as a discrete number and unit of measurement
☐	☒	A statement on whether measurements were taken from distinct samples or whether the same sample was measured repeatedly
☐	☒	The statistical test(s) used AND whether they are one- or two-sided<br>*Only common tests should be described solely by name; describe more complex techniques in the Methods section.*
☐	☒	A description of all covariates tested
☐	☒	A description of any assumptions or corrections, such as tests of normality and adjustment for multiple comparisons
☐	☒	A full description of the statistical parameters including central tendency (e.g. means) or other basic estimates (e.g. regression coefficient) AND variation (e.g. standard deviation) or associated estimates of uncertainty (e.g. confidence intervals)
☐	☒	For null hypothesis testing, the test statistic (e.g. *F*, *t*, *r*) with confidence intervals, effect sizes, degrees of freedom and *P* value noted<br>*Give P values as exact values whenever suitable.*
☒	☐	For Bayesian analysis, information on the choice of priors and Markov chain Monte Carlo settings
☒	☐	For hierarchical and complex designs, identification of the appropriate level for tests and full reporting of outcomes
☐	☒	Estimates of effect sizes (e.g. Cohen's *d*, Pearson's *r*), indicating how they were calculated

Our web collection on statistics for biologists contains articles on many of the points above.

Software and code

Policy information about availability of computer code

Data collection

No software was used for data collection

Data analysis

All data analysis was carried out using pre-existing pipelines as detailed in the Variant Calling and Classification section of the methods section of the manuscript and at https://bcbio-nextgen.readthedocs.io/en/latest/contents/pipelines.html. Source code is available at https://github.com/bcbio/bcbio-nextgen. Statistical analysis was performed using R version 4.0.0 (https://www.r-project.org/).
Whole exome sequencing data were processed with bcbio-nextgen version 1.0.9 (https://github.com/bcbio/bcbio-nextgen) with the following tools:
bcftools 1.7: https://github.com/samtools/bcftools
bedtools 2.27.1: Quinlan AR and Hall IM. BEDTools: a flexible suite of utilities for
comparing genomic features. Bioinformatics. 26(6):841–842 2010.
Biobambam 2.0.87: https://gitlab.com/german.tischler/biobambam2
bwa 0.7.17: Li H. Aligning sequence reads, clone sequences and assembly contigs with BWA-MEM. arXiv:1303.3997. 2013.
fastqc 0.11.7 https://www.bioinformatics.babraham.ac.uk/projects/fastqc/
freebayes 1.1.0.46: Garrison E, Marth G. Haplotype-based variant detection from short-read sequencing. arXiv:1207.3907. 2012.
gatk4 4.0.3.0: Van der Auwera, G.A., Carneiro, M.O., Hartl, C., Poplin, R., del Angel, G., Levy-Moonshine, A., Jordan, T., Shakir, K., Roazen, D., Thibault, J., Banks, E., Garimella, K.V., Altshuler, D., Gabriel, S. and DePristo, M.A. From FastQ Data to High-Confidence Variant Calls: The Genome Analysis Toolkit Best Practices Pipeline. Current Protocols in Bioinformatics, 43: 11.10.1-11.10.33. 2013; McKenna A, Hanna M, Banks E, et al. The Genome Analysis Toolkit: a MapReduce framework for analyzing next-generation DNA sequencing data. Genome Res. 20(9):1297-1303. 2010.
picard 2.18.2: https://broadinstitute.github.io/picard/
samtools 1.7: Li H, Handsaker B, Wysoker A, Fennell T, Ruan J, Homer N, Marth G, Abecasis G, Durbin R, and 1000 Genome Project Data Processing Subgroup, The Sequence alignment/map (SAM) format and SAMtools, Bioinformatics 25(16) 2078-9 [19505943]. 2009.
Vardict 1.5.1: Lai Z, Markovets A, Ahdesmaki M, Chapman B, Hofmann O, McEwen R, Johnson J, Dougherty B, Barrett JC, and Dry JR. VarDict: a novel and versatile variant caller for next-generation sequencing in cancer research. Nucleic Acids Res. pii: gkw227. 2016.

variant-effect-predictor 92: McLaren, W., Gil, L., Hunt, S.E. et al. The Ensembl Variant Effect Predictor. Genome Biol 17, 122. 2016. Additionally, copy number analysis was performed using GeneCN in Bio-DB-HTS version 2.10 (https://github.com/wwcrc/geneCN).

For manuscripts utilizing custom algorithms or software that are central to the research but not yet described in published literature, software must be made available to editors/reviewers. We strongly encourage code deposition in a community repository (e.g. GitHub). See the Nature Research guidelines for submitting code & software for further information.

Data

Policy information about availability of data

All manuscripts must include a data availability statement. This statement should provide the following information, where applicable:

- Accession codes, unique identifiers, or web links for publicly available datasets
- A list of figures that have associated raw data
- A description of any restrictions on data availability

The primary and processed data used to generate the analyses presented here are available via the European Genome-phenome Archive (accession EGAS00001004366) upon request to our data access committee. For more information please see https://ega-archive.org/access/data-access.
The 1000 Genomes and ExAC reference datasets can be found at http://www.internationalgenome.org (version: phase 1 SNP and InDel) and http://exac.broadinstitute.org (version ExAC.0.3.GRCh38).

Field-specific reporting

Please select the one below that is the best fit for your research. If you are not sure, read the appropriate sections before making your selection.

- [x] Life sciences
- [ ] Behavioural & social sciences
- [ ] Ecological, evolutionary & environmental sciences

For a reference copy of the document with all sections, see nature.com/documents/nr-reporting-summary-flat.pdf

Life sciences study design

All studies must disclose on these points even when the disclosure is negative.

Sample size	A case flow diagram detailing the cohort analysed is provided in the manuscript as Figure 1. Sample size was not predetermined: this study recruited all available cases in the study period. The final cohort size was 112 cases.
Data exclusions	Cases were excluded based on the criteria given in the case flow diagram provided as Figure 1. Cases were excluded based on pre-determined criteria: lack of available tumour material, WT1 positivity (indicating the sample is not EnOC), non-ovarian primary, non-EnOC ovarian carcinoma upon pathology review, concurrent metastatic malignancy, insufficient DNA quantity/quality and insufficient sequencing coverage
Replication	The manuscript describes a retrospective DNA sequencing study of a cohort of clinical cases. No replicate sequencing was performed, as is usual for this type of study. As is standard in the field, data will be made available through the European Genome-phenome Archive (see Data statement above) to allow independent analysis using the same pipeline. No replicates of immunohistochemistry were performed.
Randomization	Randomization is not applicable for retrospective cross-sectional studies, therefore no randomization was performed.
Blinding	Data collection was performed blind to the genomic analysis. Pathology review was performed blind to the genomic analysis and all other clinicopathological variables. The genomic analysis was performed blind to clinicopathological variables.

Reporting for specific materials, systems and methods

We require information from authors about some types of materials, experimental systems and methods used in many studies. Here, indicate whether each material, system or method listed is relevant to your study. If you are not sure if a list item applies to your research, read the appropriate section before selecting a response.

Materials & experimental systems

n/a	Involved in the study	
[ ]	[x]	Antibodies
[x]	[ ]	Eukaryotic cell lines
[x]	[ ]	Palaeontology
[x]	[ ]	Animals and other organisms
[ ]	[x]	Human research participants
[ ]	[x]	Clinical data

Methods

n/a	Involved in the study	
[x]	[ ]	ChIP-seq
[x]	[ ]	Flow cytometry
[x]	[ ]	MRI-based neuroimaging

Antibodies

Antibodies used	WT1 - Dako, M3561, clone 6F-H2<br>Cytokeratin 7 - CK7 - Leica, PA0138, clone RN7<br>Cytokeratin 20 - CK20 – Leica, PA0037, clone KS20.8<br>p53 - Leica, PA0057, clone DO7<br>β-catenin - Agilent, M353901-2 , clone β-Catenin-1
Validation	All of the antibodies used are utilized routinely in diagnostic pathology for human tumour diagnosis, and are therefore fully validated for use on human tissue. No other species were used in this study.<br>Immunohistochemistry (IHC) for Wilms' Tumour 1 (WT1) was performed on the Leica Bond III Autostainer using protocol F. WT1 IHC used 1:1000 dilution anti-human WT1 monoclonal mouse antibody clone 6F-H2 (DAKO). Nuclear WT1 expression in tumour cells was recorded as WT1 positive and those with complete absence of nuclear staining as WT1 negative. Positive nuclear staining of vascular endothelial cells served as internal controls.<br>Cytokeratin 7 (CK7) staining was performed using a 1:100 dilution of anti-human monoclonal mouse CK7 antibody clone RN7 (Leica). A WT1 positive high grade serous ovarian carcinoma tissue section was used as a positive control. Nuclear staining in tumour cells was considered CK7 positive.<br>Cytokeratin 20 (CK20) staining was performed using a 1:50 dilution of anti-human monoclonal mouse CK20 antibody clone KS20.8 (Leica). Normal stomach tissue was used as a positive control. Nuclear staining in tumour cells was considered CD20 positive.<br>IHC for tumour protein p53 (p53) was performed on the Leica BOND III Autostainer using protocol F. p53 IHC used a 1:50 dilution of the monoclonal mouse anti-human p53 antibody clone DO-7 (DAKO). p53 staining was recorded as aberrant (aberrant diffuse nuclear overexpression or aberrant null pattern) or wild-type (variable nuclear expression). Stromal cells served as an internal control.<br>β-catenin IHC was performed using a human tissue microarray constructed from 0.8mm cores taken from EnOC tumour regions. IHC used a 1:100 dilution of the monoclonal mouse anti-human β-catenin antibody M353901-2 (Agilent) on the Leica BOND III Autostainer. Normal tonsil tissue was used as the control. β-catenin staining was recorded as aberrant (abnormal nuclear accumulation in tumour cells) or wild-type (membranous staining only). Stromal cells served as an internal control.<br><br>Relevant citations:<br>Nakatsuka, S., Oji, Y., Horiuchi, T. et al. Immunohistochemical detection of WT1 protein in a variety of cancer cells. Mod Pathol 19, 804–814 (2006). https://doi.org/10.1038/modpathol.3800588<br>Köbel M, Reuss A, du Bois A, et al. The biological and clinical value of p53 expression in pelvic high-grade serous carcinomas. J Pathol. 2010;222(2):191-198. doi:10.1002/path.2744<br>Kim G, et al. Nuclear β-catenin localization and mutation of the CTNNB1 gene: a context-dependent association. Modern pathology. 31, 1553-1559 (2018). doi:10.1038/s41379-018-0080-0

Human research participants

Policy information about studies involving human research participants

Population characteristics	All patients with a diagnosis of endometrioid ovarian carcinoma treated at the Edinburgh Cancer Centre.
Recruitment	Patients were retrospectively identified using a local ovarian cancer patient database. Only cases with available material were considered for the study.
Ethics oversight	Ethical approval for the use of human tissue specimens for research was obtained from South East Scotland Scottish Academic Health Sciences Collaboration (SAHSC) BioResource (reference 15/ES/0094-SR494). Correlation of molecular data to clinical outcome and clinicopathological variables in ovarian cancer was approved by NHS Lothian Research and Development (reference 2007/W/ON/29). All relevant ethical regulations have been complied with, including the need for written informed consent where required.

Note that full information on the approval of the study protocol must also be provided in the manuscript.

Clinical data

Policy information about clinical studies

All manuscripts should comply with the ICMJEguidelines for publication of clinical research and a completedCONSORT checklist must be included with all submissions.

Clinical trial registration	Not applicable.
Study protocol	The manuscript reports a retrospective study, not a clinical trial. A flow diagram is provided as Figure 1 in the manuscript.
Data collection	Clinical data were derived from an ongoing clinical database housed within the Edinburgh Experimental Cancer Medicine Centre
Outcomes	Not applicable.
